# Combining metabolome and clinical indicators with machine learning provides some promising diagnostic markers to precisely detect smear-positive/negative pulmonary tuberculosis

**DOI:** 10.1186/s12879-022-07694-8

**Published:** 2022-08-25

**Authors:** Xin Hu, Jie Wang, Yingjiao Ju, Xiuli Zhang, Wushou’er Qimanguli, Cuidan Li, Liya Yue, Bahetibieke Tuohetaerbaike, Ying Li, Hao Wen, Wenbao Zhang, Changbin Chen, Yefeng Yang, Jing Wang, Fei Chen

**Affiliations:** 1 State Key Laboratory of Pathogenesis, Prevention and Treatment of High Incidence Diseases in Central Asia, Department of Respiratory Medicine, First Affiliated Hospital of Xinjiang Medical University, Urumqi, 830000 Xinjiang China; 2 Department of Respiratory Medicine, First Affiliated Hospital of Xinjiang Medical University, Urumqi, 830011 Xinjiang China; 3 CAS Key Laboratory of Genome Sciences and Information, Beijing Institute of Genomics, Chinese Academy of Sciences and China National Center for Bioinformation, Beijing, 100101 China; 4 University of Chinese Academy of Sciences, Beijing, 100049 China; 5 Department of Respiratory Medicine, Second Affiliated Hospital of Xinjiang Medical University, Urumqi, 830063 Xinjiang China; 6 State Key Laboratory of Pathogenesis, Prevention and Treatment of High Incidence Diseases in Central Asia, Urumqi, 830011 Xinjiang China; 7 Key Laboratory of Molecular Virology and Immunology, Unit of Pathogenic Fungal Infection and Host Immunity, Institute Pasteur of Shanghai, Chinese Academy of Sciences, Shanghai, 20003 China; 8 Department of Respiratory Medicine, Second Affiliated Hospital of Hainan Medical University Haikou, Hainan, 570100 China

**Keywords:** Tuberculosis (TB), *Mycobacterium tuberculosis* (*Mtb*), Smear-positive/negative pulmonary tuberculosis, Diagnostic biomarkers, Random forest, Machine learning, Metabolome, Metabolite

## Abstract

**Background:**

Tuberculosis (TB) had been the leading lethal infectious disease worldwide for a long time (2014–2019) until the COVID-19 global pandemic, and it is still one of the top 10 death causes worldwide. One important reason why there are so many TB patients and death cases in the world is because of the difficulties in precise diagnosis of TB using common detection methods, especially for some smear-negative pulmonary tuberculosis (SNPT) cases. The rapid development of metabolome and machine learning offers a great opportunity for precision diagnosis of TB. However, the metabolite biomarkers for the precision diagnosis of smear-positive and smear-negative pulmonary tuberculosis (SPPT/SNPT) remain to be uncovered. In this study, we combined metabolomics and clinical indicators with machine learning to screen out newly diagnostic biomarkers for the precise identification of SPPT and SNPT patients.

**Methods:**

Untargeted plasma metabolomic profiling was performed for 27 SPPT patients, 37 SNPT patients and controls. The orthogonal partial least squares-discriminant analysis (OPLS-DA) was then conducted to screen differential metabolites among the three groups. Metabolite enriched pathways, random forest (RF), support vector machines (SVM) and multilayer perceptron neural network (MLP) were performed using Metaboanalyst 5.0, “caret” R package, “e1071” R package and “Tensorflow” Python package, respectively.

**Results:**

Metabolomic analysis revealed significant enrichment of fatty acid and amino acid metabolites in the plasma of SPPT and SNPT patients, where SPPT samples showed a more serious dysfunction in fatty acid and amino acid metabolisms. Further RF analysis revealed four optimized diagnostic biomarker combinations including ten features (two lipid/lipid-like molecules and seven organic acids/derivatives, and one clinical indicator) for the identification of SPPT, SNPT patients and controls with high accuracy (83–93%), which were further verified by SVM and MLP. Among them, MLP displayed the best classification performance on simultaneously precise identification of the three groups (94.74%), suggesting the advantage of MLP over RF/SVM to some extent.

**Conclusions:**

Our findings reveal plasma metabolomic characteristics of SPPT and SNPT patients, provide some novel promising diagnostic markers for precision diagnosis of various types of TB, and show the potential of machine learning in screening out biomarkers from big data.

**Supplementary Information:**

The online version contains supplementary material available at 10.1186/s12879-022-07694-8.

## Background

According to WHO reports, tuberculosis (TB) caused by *mycobacterium tuberculosis* (*Mtb*) had been the leading lethal infectious disease worldwide for a long time (2014–2019) until the COVID-19 global pandemic (2020–2021) [1], and there were ∼ 10 million new TB cases every year [2, 3]. According to the data collected from the National Notifiable Disease Reporting System (NNDRS), the annual incidence of Xinjiang is 169.05/100,000 and the mean annual rate of reported PTB (pulmonary tuberculosis) in Kashgar was 450.91/100,000 from 2011 to 2020 [4]. Why are there so many TB patients and death cases around the world? One reason is because of the difficulties in precise diagnosis of TB, especially for some smear-negative pulmonary tuberculosis (SNPT) cases that usually show similar symptoms to other lung diseases [5, 6]. In some countries/regions, SNPT patients even account for more than 50% of all TB cases [7].

At present, although three common methods (sputum-smear microscopy, sputum culture tests and Xpert MTB/RIF assays) can achieve relatively precise diagnosis for most TB patients, they still have some disadvantages (such as relatively low sensitivity for sputum-smear microscopy, time-consuming for sputum culture, and relatively high cost for Xpert), further leading to some false negative/positive cases [1, 6, 8–10]. The failure diagnosis may result in delayed treatment, poor therapeutic effect and higher treatment costs [11, 12]. Nowadays, how to timely and accurately detect various types of TB remains a substantial challenge for global TB control.

The rapid development of various omics technologies offers a great opportunity for precision diagnosis of various types of diseases [13–16]. Among them, metabolome has been widely applied in biomarker discovery for the detection, diagnosis and treatment of various diseases, since they have been reported to be closely associated with disease genotypes and phenotypes [17]. In the TB research field, Deng et al. reported significantly changed glutathione and histamine in the urine of active TB patients, which could distinguish them from latent tuberculosis infected patients [18]; Huang et al.provided some potential plasma metabolite biomarkers (Xanthine, 4-Pyridoxate, and d-glutamic acid) for TB diagnosis [19]; Sun et al.revealed some potential metabolite biomarkers for pediatric TB diagnosis by l-valine, pyruvic acid and betaine in plasma [20]. However, the metabolite biomarkers for precision diagnosis of smear-positive and smear-negative tuberculosis (SPPT and SNPT) remain to be uncovered.

In our study, we performed plasma metabolomic analyses from 27 SPPT patients, 37 SNPT patients and 36 controls. Metabolomic profiling revealed dysfunctional fatty acid and amino acid metabolisms in SPPT and SNPT patients. Four optimized diagnostic biomarker combinations (two lipid/lipid-like molecules and seven organic acids/derivatives, and one clinical indicator) were then screened out for precise diagnosis of SPPT and SNPT patients and controls through the random forest (RF). The classification performance of the four combinations was further verified by other two machine learning methods: support vector machines (SVM) and multilayer perceptron neural network (MLP). Our findings revealed the metabolomic characteristics of SPPT and SNPT patients, provided some promising diagnostic markers for precision diagnosis of various types of TB patients, and showed the potential of machine learning in the detection of diagnostic biomarkers.

## Methods

### Study participates

In our study, all the TB patients (including 27 SPPT and 37 SNPT patients) were recruited from the Tuberculosis Prevention and Treatment Institute of Kashgar, the Second People’s Hospital of Aksu, and the Kuqa County Infectious Disease Hospital during October 2017 to October 2018. 36 control people (Ctrl) without TB infection from the First Affiliated Hospital of Xinjiang Medical University were also enrolled (Table 1, Additional file 1: Fig. S1). The diagnosis of TB was based on clinical symptoms and microbiological evidence according to Diagnosis for Pulmonary Tuberculosis (WS 288-2017). SPPT patients were diagnosed when one of the following microbiological evidence was obtained: (1) positive stain for acid-fast bacilli, (2) positive culture for *Mtb*, (3) positive Xpert test. SNPT patients were diagnosed based on the classical clinical symptoms although acid-fast bacilli were negative. The exclusion criteria included: (1) the TB patients in treatment period; (2) the TB patients with other chronic or acute diseases such as pregnancy complications, cardiac dysfunction, renal disease, psychiatric disease, gastrointestinal disease, uncontrolled hypertension, and some severe stress states (including cardiovascular and cerebrovascular events, severe infection, traumatic surgery, and severe wasting diseases). This study was approved by the Ethical Committee of First Affiliated Hospital of Xinjiang Medical University (20171123-06-1908A).

**Table 1:** Baseline characteristics of SPPT and SNPT patients

	Total (N = 100)	SPPT (N = 27)	SNPT (N = 37)	Control (N = 36)	^*^Adjusted *p-value* (SPPT/Ctrl)	^*^Adjusted *p-value* (SNPT/Ctrl)	^*^Adjusted *p-value* (SPPT/SNPT)	χ^2^, F or H value (SPPT/SNPT/Ctrl)	*p-value* (SPPT/SNPT/Ctrl)
Gender (%)					0.162	0.43	0.449	4.868^$^	0.088
Male	52 (52.0)	18 (66.7)	20 (54.1)	14 (38.9)					
Female	48 (48.0)	9 (33.3)	17 (45.9)	22 (61.1)					
Age (years, median [Q1–Q3])	53.50 (35.00–67.25)	51.00 (32.50–71.00)	60.00 (49.00–71.00)	43.50 (34.00–59.25)	0.087	0.011	0.315	6.369	0.041
Occupations (%)					–	–	0.934	–	–
Farmer	54 (54.0)	23 (85.2)	31 (83.8)	0 (0.0)					
Retiree	11 (11.0)	1 (3.7)	1 (2.7)	9 (25.0)					
Student	4 (4.0)	1 (3.7)	3 (8.1)	0 (0.0)					
Other	6 (6.0)	2 (7.4)	2 (5.4)	2 (5.6)					
(Missing value)	25 (25.0)	0 (0.0)	0 (0.0)	25 (69.4)					
Marital status (%)					–	–	0.183	–	–
Single	7 (7.0)	5 (18.5)	2 (5.4)	0 (0.0)					
Married	92 (92.0)	22 (81.5)	35 (94.6)	35 (97.2)					
(Missing value)	1 (1.0)	0 (0.0)	0 (0.0)	1 (2.8)					
BMI (kg/m^2^, mean[SD])	23.19 (4.44)	20.22 (3.95)	22.82 (3.93)	25.33 (4.13)	< 0.001	0.022	0.027	10.02^#^	< 0.001
Smoking status (%)					–	–	–	–	–
Never	79 (79.0)	24 (88.9)	34 (91.9)	21 (58.3)					
Current	11 (11.0)	3 (11.1)	2 (5.4)	6 (16.7)					
Former	1 (1.0)	0 (0.0)	1 (2.7)	0 (0.0)					
(Missing value)	9 (9.0)	0 (0.0)	0 (0.0)	9 (25.0)					
Drinking status (%)					–	–	–	–	–
Never	80 (80.0)	25 (92.6)	37 (100.0)	18 (50.0)					
Current	11 (11.0)	2 (7.4)	0 (0.0)	9 (25.0)					
Former	1 (1.0)	0 (0.0)	0 (0.0)	1 (2.8)					
(Missing value)	8 (8.0)	0 (0.0)	0 (0.0)	8 (22.2)					
TB contact (%)					–	–	< 0.001	–	–
Yes	44 (68.8)	8 (29.6)	36 (97.3)	–					
No	16 (25.0)	16 (59.3)	0 (0.0)	–					
(Missing value)	4 (6.3)	3 (11.1)	1 (2.7)						
TB treatment (%)					–	–	0.836	–	–
New cases of TB	17 (26.6)	7 (25.9)	10 (27.0)	–					
Previously treated	41 (64.1)	14 (51.9)	27 (73.0)	–					
(Missing value)	6 (9.4)	6 (22.2)	0 (0.0)						
Cavitary pulmonary TB (%)	33 (51.6)	19 (70.4)	14 (37.8)	–	–	–	0.02		–
Symptoms (%)									
Cough	60 (92.3)	22 (81.5)	37 (100.0)	–	–	–	0.024	–	–
Expectoration	60 (92.3)	22 (81.5)	37 (100.0)	–	–	–	0.024	–	–
Dyspnea	35 (54.7)	6 (22.2)	29 (78.4)	–	–	–	< 0.001	–	–
Chest discomfort	13 (20.3)	5 (18.5)	8 (21.6)	–	–	–	1	–	–
Fever	5 (7.8)	3 (11.1)	2 (5.4)	–	–	–	0.713	–	–
Hemoptysis	2 (3.1)	1 (3.7)	1 (2.7)	–	–	–	1	–	–
Chest pain	2 (3.1)	1 (3.7)	1 (2.7)	–	–	–	1	–	–
Nausea	1 (1.6)	0 (0.0)	1 (2.7)	–	–	–	-	–	–
Fatigue	2 (3.1)	2 (7.4)	0 (0.0)	–	–	–	0.174	–	–
Night sweats	1 (1.6)	0 (0.0)	1 (2.7)	–	–	–	–	–	–
Short of breath	3 (4.7)	3 (11.1)	0 (0.0)	–	–	–	–	–	–

*BMI* body mass index, Data are shown as n (%), mean (SD) or median (Q1–Q3). *p-values* are calculated after exclusion of missing data for that variable; *Adjusted p-value for multiple comparisons using Bonferroni-Holm correction. SD: standard deviation; (Q1–Q3): 25th Quartile–75th Quartile. ^$^Chi aquare test; ^#^One Way ANOVA;

### Plasma sample preparation

A total of 0.5–1 mL of the whole blood sample from each participant was collected by cubital vein phlebotomy using a heparin anticoagulation collection tube. The blood samples were then centrifuged for 10 min (1500 rpm/min, 4 °C) to remove the blood cells, and the supernatants were immediately frozen in liquid nitrogen and stored at − 80 °C until use. Frozen plasma samples were slowly thawed at 4 °C, and each 100 μL aliquot was mixed with 400 μL of pre-cooled methanol/acetonitrile (1:1, v/v) solution. After the vortex, the mixture was incubated at − 20 °C for 10 min, and then centrifuged for 15 min (14,000 rcf, 4 °C). The supernatants were freeze-dried and reconstituted in 100 μL acetonitrile/water (1:1, v/v) solution for LC–MS/MS analysis (Shanghai Applied protein technology Co., Ltd, Shanghai, China)**.**

### Metabolite measurement

Metabolites were extracted from plasma samples. Untargeted metabolomics analysis was conducted by using ultra-high-performance liquid chromatography (UHPLC, 1290 Infinity LC, Agilent Technologies, Palo Alto, CA, USA) and a quadrupole time-of-flight mass spectrometer (TripleTOF 6600; AB Sciex, Framingham, MA, USA). The separation was performed using a 2.1 mm × 100 mm ACQUITY UPLC BEH 1.7 μm column (Waters, Wexford, Ireland). The mobile phase consisted of A. 25 mM ammonium acetate with 25 mM ammonium hydroxide; B. acetonitrile. Gradient elution was performed as follows: 95% B for 0.5 min, and was reduced linearly to 65% in 7 min, next, the gradient was reduced to 40% in 2 min, increased to 95% in 0.1 min, then with a re-equilibration period employed for 3 min. The flow rate was set to 0.3 mL min^−1^, column temperature at 25 °C and injection volume of 2 µL. The ESI conditions were as follows: Ion Source Gas1(Gas1): 40 psi; Ion Source Gas2 (Gas2): 80 psi; curtain gas (CUR): 30 psi; source temperature: 650℃; IonSpray Voltage Floating (ISVF) ± 5500 V. The raw data were converted to MzXML by MSconventer (ProteoWizard, Palo Alto, CA, USA), and imported into XCMS software (Scripps Research Institute, La Jolla, CA, USA) for alignment, feature detection, retention time correction, and data filtering.

### Bioinformatics analysis

Multivariable analysis was conducted using SIMCA-P software (version 14.1 Umetrics, Umea, Sweden). The orthogonal partial least squares-discriminant analysis (OPLS-DA, Umetrics, Umea, Sweden) was then performed to screen the differential metabolites, and the robustness of the OPLS-DA model was evaluated by using the sevenfold cross-validation and response permutation testing. Differentially abundant metabolites (DAMs) were confirmed based on variable importance in projection (VIP) > 1 obtained from the OPLS-DA model and Student’s t-test p values (*p* < 0.05). The chemical taxonomy of DAMs was determined according to “The Human Metabolome Database (HMDB)” (https://hmdb.ca/). Metabolite enriched pathway analysis was implemented with the online software of Metaboanalyst 5.0 [21].

### Data preprocessing

After removing the indicators with a large proportion of missing values (≥ 20%, for details see Additional file 1: Table S1), 24 remaining clinical indicators and 96 DAMs were included to screen out potential diagnostic biomarkers. Categorical variables were then coded with dummy variables. A total of 100 individuals (27 SPPT patients, 37 SNPT patients and 36 controls) were then randomly separated into a training set (n = 81) and a test set (n = 19) using createDataPartition function in R caret package (http://topepo.github.io/caret/data-splitting.html). Further K-Nearest Neighbor was adopted to impute the missing values of the remaining indicators [22]. Specifically, a KNN model (http://topepo.github.io/caret/pre-processing.html) was created based on the training set, which was then applied to predict the missing values in the test set. As a result, the standardized data sets were obtained. Principal component analysis (PCA) was then applied to detect global clinical indicators and metabolic alterations among different samples [23]. Pearson correlation coefficients among the clinical indicators and DAMs were calculated by the findCorrelation function in R software (https://github.com/topepo/caret/blob/master/pkg/caret/R/findCorrelation.R). The features with high mean absolute correlations (≥ 0.7) were excluded (Additional file 2).

### Biomarker detection and verification using three machine learning methods (RF, SVM and MLP)

First, the pre-select 20 clinical indicators and 58 identified DAMs (78 features, defined as F_0_ set) were included for the classification of SPPT/Ctrl, SNPT/Ctrl, SPPT/SNPT and SPPT/SNPT/Ctrl groups. RF was then adopted to evaluate the classification performance of the F_0_ set. AUCs were calculated by receiver operating characteristic (ROC) analysis using the roc () function of pROC package in R [24].

We then used recursive feature elimination (R package caret) to decrease the number of features in the RF model (parameter use "rfFuncs” and “cv”) [25]. Mean decrease in Gini coefficient (MDG) was further used for measuring variable importance, and the combinations of important features with accuracy over 90% were finally selected for machine learning. Here, the selected features in SPPT/Ctrl, SNPT/Ctrl, SPPT/SNPT and SPPT/SNPT/Ctrl groups were defined as F_1_, F_2_, F_3_ and F_4_, respectively. Ultimately, the classification accuracies of the above four feature sets were verified by other two machine learning methods: SVM and MLP. The SVM was realized using “e1071” R package. The MLP classification algorithm including two hidden layers (each layer consists of 15 nodes) was completed using the “Tensorflow” package of Python [26]. To avoid overfitting, tenfold cross-validation (CV) was employed on the train set, which was further randomly split into 90% for “actual train set” and 10% for “validation set” for ten times. Ultimately, the test sets were used to evaluate the accuracy, sensitivity, specificity, positive predictive value (PPV) and negative predictive value (NPV) of each trained model. The codes were deposited on GitHub (https://github.com/ChenF-Lab/SPPT.git).

### Statistical analysis

The continuous variables were described using mean (standard deviation), median and interquartile ranges (Q1–Q3). The categorical variables were described as frequency rates and percentages. Independent samples t-test was used for comparing means of normally distributed variables while Mann Whitney U test for not normally distributed variables. One-Way ANOVA or Kruskal Wallis test were used to compare variables among three groups. Categorical variables were compared using the chi-square test. Bonferroni-Holm correction was applied to obtain the corrected p-value for multiple comparisons. All the statistical analyses were performed using R software (version 4.0.2; an open-source free software). Two-sided p values of less than 0.05 were considered statistically significant.

## Results

### Demographics and clinical characteristics of the SPPT and SNPT patients

In our study, 64 TB patients, including 27 SPPT patients and 37 SNPT patients, were enrolled to identify the biomarker candidates for tuberculosis diagnosis. 36 non-TB individuals were also included as controls. Here, the majority of TB patients are males (59.4%), and more than 80% of TB patients are farmers. The median age of SNPT patients was 60.0 years old (Q1–Q3: 49.00–71.00), which was significantly higher than that of SPPT patients (51.0 years old, Q1–Q3: 32.50–71.00) and controls (43.5 years old, Q1–Q3: 34.00–59.25). The mean BMIs of SPPT and SNPT patients were 20.22 kg/m^2^ (SD: 3.95) and 22.82 kg/m^2^ (SD: 3.93), respectively, which were significantly lower than controls (*p* < 0.001). The common symptoms were cough (92.3%) and expectoration (92.3%), followed by dyspnea (54.7%) and chest discomfort (20.3%). Notably, 70.4% of SPPT patients belong to cavitary pulmonary TB which has been previously demonstrated to be associated with higher bacterial load [27] (Table 1).

Clinical characteristic analysis showed significantly decreased albumin and serum creatinine, and increased erythrocyte sedimentation rate (ESR) for the TB patients (Table 2). Here, the albumin of SPPT patients was significantly lower than that of SNPT patients (SPPT: 35.30 g/L; SNPT: 39.20 g/L; adjusted *p* = 0.002), indicating more serious chronic inflammation/malnutrition for the SPPT patients [28, 29]; the serum creatinine was significantly lower in TB patients compared with controls, but showed no difference between SPPT and SNPT patients, suggesting renal injury induced by tuberculous drugs; the ESR of SPPT patients (67.50 mm/h) was significantly higher than that of SNPT patients (43.00 mm/h), and ESR had been reported to identify active tuberculosis and differentiate pulmonary tuberculosis from bacterial community-acquired pneumonia [30].

**Table 2:** Clinical indicators of SPPT and SNPT patients

	Normal range	SPPT		SNPT		Control		^*^Adjusted *p-value* (SPPT/Ctrl)	^*^Adjusted *p-value* (SNPT/Ctrl)	^*^Adjusted *p-value* (SPPT/SNPT)	H value (SPPT/SNPT/Ctrl)	*p-value* (SPPT/SNPT/Ctrl)
		Patients	Median (Q1–Q3)	Patients	Median (Q1–Q3)	Patients	Median (Q1–Q3)					
Blood routine												
Leucocytes, × 10^9^/L	3.5–9.5	27	7.20 (5.89–8.61)	37	7.65 (6.32–8.74)	36	6.49 (5.66–7.54)	0.009	0.032	0.413	6.86	0.032
Neutrophils, × 10^9^/L	1.8–6.3	10	7.83 (4.62–11.61)	37	5.10 (3.92–5.94)	36	3.82 (3.18–4.51)	< 0.001	< 0.001	0.068	19.6	< 0.001
Erythrocytes, × 10^12^/L	4.3–5.8	27	4.28 (3.88–4.66)	37	4.78 (4.35–4.98)	36	4.77 (4.46–5.25)	< 0.001	0.682	0.003	14.06	0.001
Hemoglobin, g/L	130–175	27	117.00 (104.50–134.00)	37	139.00 (132.00–151.00)	36	140.00 (132.00–151.50)	< 0.001	0.961	< 0.001	29.01	< 0.001
Platelets, × 10^9^/L	125–300	26	305.50 (238.50–371.75)	37	225.00 (201.00–279.00)	36	265.00 (234.50–348.25)	0.15	0.003	0.003	10.31	0.006
Eosinophils, × 10^9^/L	0.02–0.52	27	0.12 (0.04–0.34)	37	0.15 (0.10–0.22)	36	0.11 (0.07–0.24)	0.12	0.174	0.12	0.61	0.739
Basophils, × 10^9^/L	0–0.06	27	0.02 (0.00—0.07)	37	0.02 (0.01–0.05)	36	0.02 (0.01—0.02)	< 0.001	0.005	< 0.001	0.73	0.693
Blood biochemistry												
Total protein, g/L	65–85	27	66.50 (58.75–0.30)	37	66.30 (62.70–68.70)	34	73.40 (70.93–77.18)	< 0.001	< 0.001	0.402	31.68	< 0.001
Albumin, g/L	40–55	27	35.30 (31.00–38.75)	37	39.20 (36.00–43.00)	34	44.59 (42.73–45.66)	< 0.001	< 0.001	0.002	47.21	< 0.001
Globulin, g/L	20–40	16	30.65 (26.00–35.75)	37	26.40 (24.00–29.30)	34	28.53 (26.36–32.54)	0.189	0.087	0.043	7.63	0.022
Triglyceride, mmol/L	0.5–1.9	25	0.99 (0.80–1.22)	35	1.14 (0.89–1.60)	35	1.08 (0.76–1.63)	0.83	0.696	0.696	1.8	0.408
Total cholesterol, mmol/L	2.3–5.2	25	3.45 (2.87–3.69)	35	3.63 (3.27–4.58)	35	4.15 (3.76–4.98)	< 0.001	0.048	0.302	12.12	0.002
ASP, IU/L	9–60	27	23.00 (18.00–36.50)	37	21.00 (18.00–31.00)	35	19.80 (17.65–22.30)	0.014	0.043	0.508	3.75	0.153
ALT, IU/L	9–50	27	21.50 (12.55–43.15)	37	19.00 (15.00–29.00)	35	24.20 (15.20–30.40)	0.269	0.269	0.586	0.51	0.776
AKP, IU/L	45–125	27	68.00 (53.65–102.50)	37	98.00 (74.00–129.00)	32	76.85 (65.80–89.80)	0.155	0.005	0.005	10.13	0.006
γ-GT, IU/L	10–60	27	33.70 (22.00–55.00)	37	26.00 (20.00–59.00)	32	21.50 (15.75–33.50)	0.189	0.189	0.559	3.79	0.15
Creatinine, μmol/L	57–97	27	55.20 (44.80–70.70)	37	54.00 (47.00–72.00)	36	65.77 (60.15–74.81)	0.025	0.022	0.961	7.93	0.019
Total bilirubin, μmol/L	0–26	27	9.60 (7.90–11.85)	37	11.93 (9.20–19.60)	34	12.13 (10.27–13.95)	0.042	0.123	0.042	6.61	0.037
Direct bilirubin, μmol/L	0–8	27	3.39 (2.30–4.50)	37	2.18 (0.30–3.30)	6	2.31 (2.12–3.82)	0.421	< 0.001	< 0.001	6.48	0.039
Indirect bilirubin, μmol/L	0–14	15	6.30 (4.23—8.37)	37	7.90 (6.11–12.15)	6	6.40 (6.20–6.55)	0.402	0.115	0.115	4.53	0.104
Inflammatory-related biomarkers												
ESR, mm/h	0–15	26	67.50 (43.75–94.25)	35	43.00 (14.00–62.00)	0	–	–	–	0.003		–
C-reaction protein, mg/L	0–4	27	16.53 (9.69–65.08)	33	1.67 (0.80–3.67)	0	–	–	–	< 0.001		–
Procalcitonin, ng/mL	0–0.05	15	0.10 (0.07–0.36)	34	0.02 (0.01—0.11)	0	–	–	–	0.013		–

Data are shown as median (Q1–Q3); Missing data of variables are omitted here and showed in the Additional file 1: Table S1; *Adjusted p-value for multiple comparisons using Bonferroni-Holm correction. SD: standard deviation; (Q1–Q3): 25th Quartile—75th Quartile. ASP: aspartate aminotransferase; ALT: alanine aminotransferase; AKP: Alkaline phosphatase; γ-GT: γ-glutamyl transpeptidase; ESR: Erythrocyte sedimentation rate

Additionally, neutrophils, C-reactive protein and procalcitonin were significantly upregulated in SPPT patients than in SNPT ones, while the hemoglobin of SPPT patients was significantly downregulated than that of SNPT ones. These indicators were all in the normal range for the SNPT patients, reflecting stronger immune and inflammatory reactions of SPPT patients.

### Plasma metabolomic analysis showing dysfunctional fatty acid and amino acid metabolisms in SPPT and SNPT patients

**Fig. 1:**
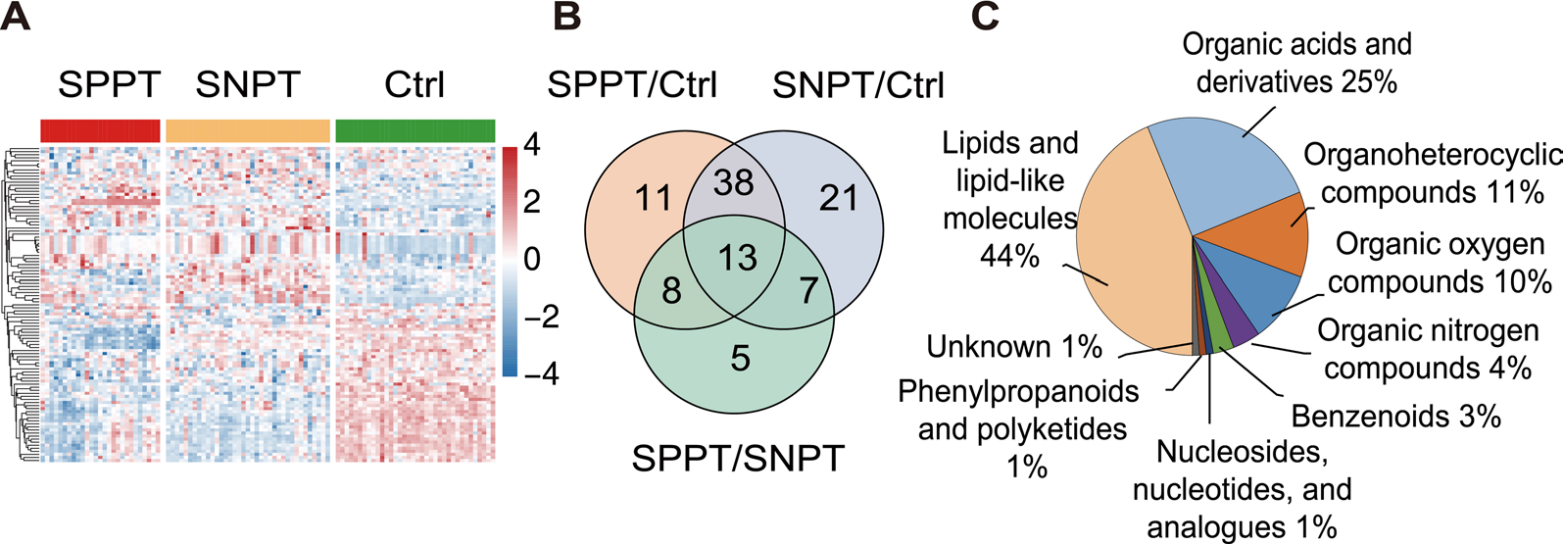
Plasma metabolomic analysis for the SPPT patients, SNPT patients and controls. **A** Heatmap showing 103 differential abundant metabolites (DAMs, VIP > 1, p < 0.05) among the three groups. The colored bar above the heatmap represent the SPPT (red), SNPT (orange) and Ctrl (green) samples. The color key indicates the scaled expression levels of the 103 metabolites for the three groups. **B** Venn diagram showing the differential metabolites among the three groups. **C** Pie chart showing the chemical classification of the 103 significantly differentially abundant metabolites according to the HMDB database

Metabolome analysis was performed on the plasma samples from SPPT, SNPT and Ctrl groups, and a total of 103 DAMs were identified (Fig. 1A, B and Additional files 3, 4, 5). The heatmap showed the DAM expression profiles for the three groups, and the metabolomic profiling of SPPT patients was more similar to that of SNPT patients rather than controls (Fig. 1A). We then classified all DAMs into nine categories based on their chemical taxonomy according to “The Human Metabolome Database” (https://hmdb.ca/), including “Lipids and lipid-like molecules” (~ 44%), “Organic acids and derivatives” (~ 25%), “Organoheterocyclic compounds” (12%) and “Organic oxygen compounds” (~ 10%) (Fig. 1C).

In the SPPT/Ctrl group, 70 DAMs were identified, most of which were lipids/lipid-like molecules (31) and organic acids/derivatives (16) (Additional file 3). Compared with controls, 77% (24/31) of the lipids/lipid-like molecules (19 fatty acyls, 3 glycerophospholipids, etc.) and 81.5% (13/16) of the organic acids/derivatives showed significantly down-regulated trend (FC < 1, *p* < 0.05) in the SPPT group, indicating the dysfunctional lipid and amino acid metabolisms in the SPPT patients as previously reported [31, 32].

In the SNPT/Ctrl group, 79 DAMs were obtained, most of which also belonged to lipid/lipid-like molecules (37, top-1) and organic acids/derivatives (16, top-2) (Additional file 4). Compared to controls, 73% (27/37) of lipids/lipid-like molecules and 56% (9/16) organic acids/derivatives showed significantly down-regulated trend in the SNPT samples, also indicating the dysfunctional lipid and amino acid metabolisms in the SNPT patients.

In the SPPT/SNPT group, 33 DAMs were identified, most of which also belonged to lipid/lipid-like molecules (17) and organic acids/derivatives (10) (Additional file 5): 53% (9/17) of the lipid/lipid-like molecules were significantly downregulated (4 fatty acyls, 2 glycerophospholipids, 2 prenol lipids, etc.), and 47% (8/17) of them were significantly up-regulated (5 fatty acyls and 3 steroids/steroid derivatives); 90% (9/10) of the organic acids/derivatives were significantly down-regulated (eight carboxylic acids and derivatives and one organic carbonic acid/derivative), and only one was significantly up-regulated (hydroxy acid/derivative).

In all, the three groups (SPPT/Ctrl, SPPT/Ctrl and SPPT/SNPT) showed significant enrichments in lipids/lipid-like molecules (top-1) and organic acids/derivatives (top-2).

**Fig. 2:**
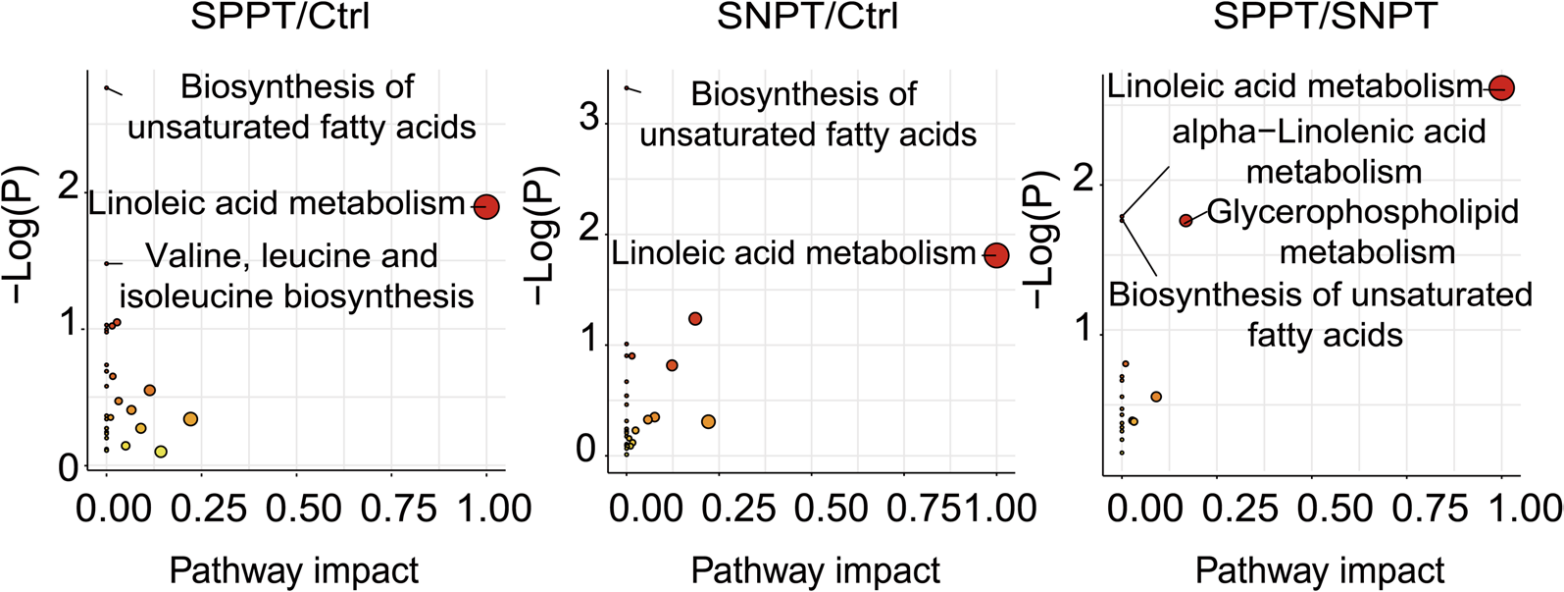
Scatter plots showing the significantly enriched metabolic pathways among the three groups. The size and color of circles indicate the impact score and *p*-value of the enriched pathways, respectively

To evaluate the metabolic characteristics of the three groups, we further performed the pathway analysis for these DAMs using MetaboAnalyst 5.0. The results showed significantly differential enrichment of lipid and amino acid metabolism related pathways among the three groups (Fig. 2 and Additional file 1: Table S2–S4). In the SPPT/Ctrl group, the DAMs were significantly enriched in two fatty acid metabolism related pathways (“Biosynthesis of unsaturated fatty acids pathway”, “Linoleic acid metabolism pathway”) and one amino acid metabolism related pathway (“Valine, leucine and isoleucine biosynthesis pathway”), indicating significantly unregulated unsaturated fatty acid and amino acid metabolisms in the SPPT samples as previously reported [32–35]. In the SNPT/Ctrl group, the DAMs were significantly enriched in the same two fatty acid-related pathways as those in the SPPT/Ctrl group. In the SPPT/SNPT group, four lipid-related metabolic pathways, including “Linoleic acid metabolism pathway”, “Glycerophospholipid metabolism pathway”, “alpha-Linolenic acid metabolism pathway” and “Biosynthesis of unsaturated fatty acids pathway”, were significantly enriched, indicating more serious dysfunction of fatty acid metabolisms in the SPPT patients than in the SNPT patients. Overall, the two significant enrichment unsaturated fatty acid metabolism related pathways were shared by the three groups (SPPT/Ctrl, SNPT/Ctrl and SPPT/SNPT), indicting similar dysfunctional fatty acid metabolisms among the three groups; they should be associated with disease progress of TB.

Taken together, the above results showed the dysfunctions of fatty acid and amino acid metabolisms in the SPPT and SNPT patients, where these dysfunctions in the SPPT patients were more serious than those in the SNPT patients.

### Precise classification among the three groups using DAMs and clinical indicators

**Fig. 3:**
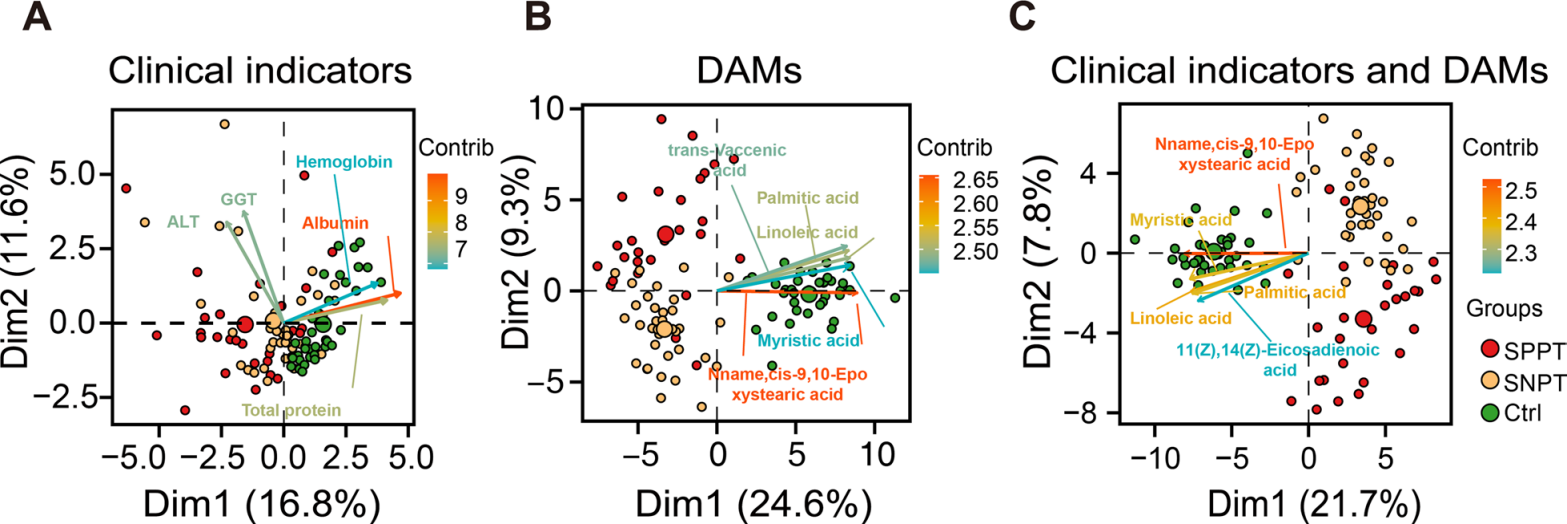
Principal component analyses of clinical indicators (**A**), DAMs (**B**) and their combination (**C**) among SPPT, SNPT and controls. The color key indicates the contribution of the top 5 variables from high (reddish arrows) to low contribution (bluish arrows)

We then investigated the classification effect for the three groups (SPPT, SNPT patients and controls) using all the practicable clinical laboratory indicators (24) and DAMs (96). Here, seven drug-related metabolites (Dehydroabietic acid, Dyphylline, EDTA, Levofloxacin, Norethindrone Acetate, Sunitinib and Thioetheramide-PC) were excluded to increase the general applicability of classification according to HMDB database [36, 37]. PCA analysis was first applied to explore whether clinical indicators and DAMs could be used to distinguish the SPPT, SNPT and Control samples (Fig. 3): DAMs displayed obvious separation while clinical indicators showed poor separation among the three groups; clinical indicators combined with DAMs showed the best classification performance among the three groups. Here the top ten contributed variables of PC1 and PC2 are all belong to DAMs, indicating a greater contribution of DAMs than clinical indicators (Additional file 1: Fig. S2).

All the 120 features (24 clinical indicators and 96 DAMs) were further calculated for correlation coefficients between pairwise features (Additional file 2). 42 features were excluded due to their higher mean absolute correlation coefficients (≥ 0.7), and the remaining 78 features were denoted as F_0_ set for classification analysis among the three groups. RF and ROC analyses were then used to evaluate the classification performance of the 78 features for the SPPT/Ctrl, SNPT/Ctrl, SPPT/SNPT and SPPT/SNPT/Ctrl groups. The results showed the tenfold cross-validation average accuracy of 98% (SD: 0.06), 100% (SD: 0.00) and 92% (SD: 0.09) for the binary classifications of the SPPT/Ctrl, SNPT/Ctrl and SPPT/SNPT groups in validation sets, respectively (Additional file 1: Table S5). Further, 100% accuracy (AUC: 1.00) was obtained for all the binary classifications of in test sets (Additional file 1: Fig. S3). For the three-class classification of SPPT/SNPT/Ctrl group, the 78 features also showed good classification performance in validation sets (average accuracy: 95% (SD: 0.09) and test set (accuracy: 94.74%; sensitivity: 80%, 100% and 100% for SPPT, SNPT and control groups; and specificity: 100%, 91.67%, and 100% for SPPT, SNPT and control groups; PPV: 100%, 87.50% and 100% for SPPT, SNPT and control groups; NPV: 93.33%, 100% and 100% for SPPT, SNPT and control groups;). These indicated the precise classification among the SPPT and SNPT patients and controls using the combination of clinical indicators and DAMs (F_0_).

### Selecting the optimized biomarker combinations to precisely identify any one of the SPPT and SNPT patients and controls.

**Fig. 4:**
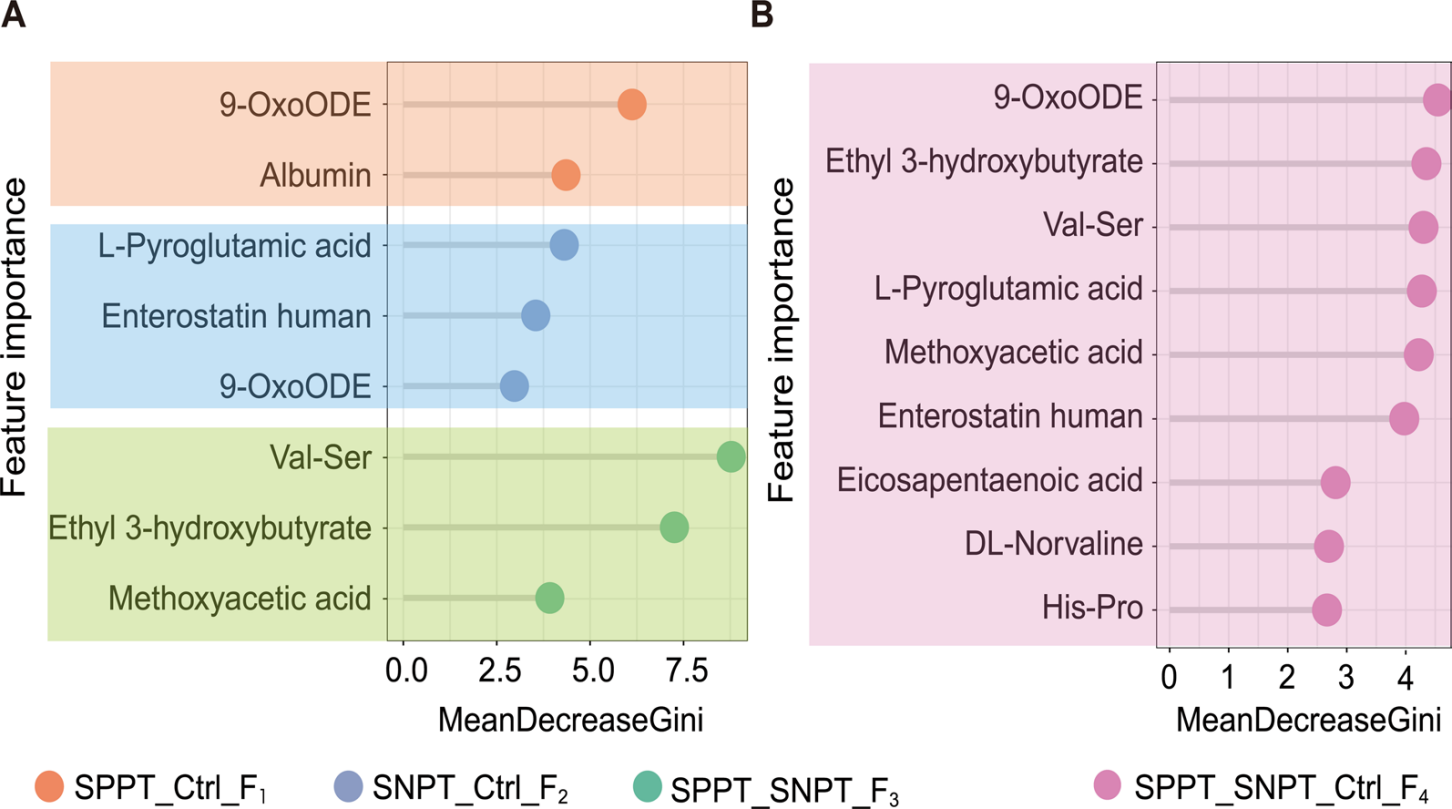
Importance of the screened features for identifying SPPT, SNPT patients from controls. **A** Importance of the clinical and metabolic features from different optimized combinations for precisely binary classification of SPPT/Ctrl, SNPT/Ctrl and SPPT/SNPT groups (from top to bottom) using random forest model. **B** Importance of the clinical and metabolic features from the four optimized combinations for simultaneous classification of SPPT, SNPT and Ctrl groups

To explore the optimized diagnostic biomarker combinations, we then evaluated the contribution of features to the classification using random forest algorithm. The results revealed the optimized biomarker combinations with higher accuracy (> 0.9, Additional file 1: Fig. S4) for precision binary and three-class classifications among the three groups in training sets, including a two biomarker combination (albumin and 9-OxoODE, defined as “F_1_ set”) for precisely distinguishing SPPT from controls, a three biomarker combination (L-Pyroglutamic acid (PGA), Enterostatin human and 9-OxoODE, defined as “F_2_ set”) for precisely differentiating SNPT from controls, a three biomarker combination (Val-Ser, Methoxyacetic acid (MAA) and Ethyl 3-hydroxybutyrate, defined as “F_3_ set”) for precisely distinguishing SPPT from SNPT, and a nine biomarker combination (9-OxoODE, PGA, Val-Ser, Ethyl 3-hydroxybutyrate, MAA, Enterostatin human, DL-Norvaline, His-Pro and Eicosapentaenoic acid (EPA), defined as “F_4_ set”) for simultaneously precise identification of SPPT and SNPT patients and controls (Fig. 4, Additional file 1: Table S6).

**Fig. 5:**
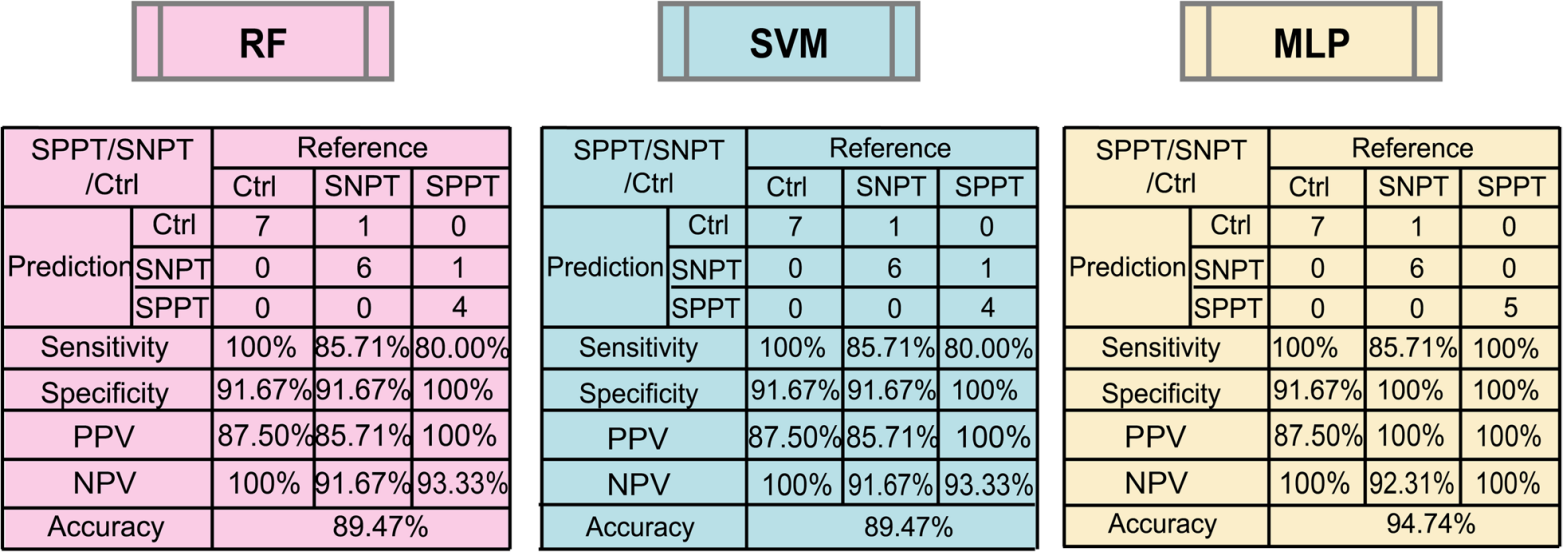
Confusion matrixes for discriminating SPPT, SNPT and controls with F_4_ set in the test sets. Confusion matrixes from left to right show the classification performance of SPPT/SNPT/Ctrl groups in the test sets using RF, SVM and MLP models, respectively. F_4_ set: 9-OxoODE, PGA, Val-Ser, Ethyl 3-hydroxybutyrate, MAA, Enterostatin human, DL-Norvaline, His-Pro and Eicosapentaenoic acid

The binary classification performance of the above biomarker combinations (F_1_, F_2_ and F_3_) was further verified in test sets with high accuracy, sensitivity and specificity (accuracy: 83.33% for SPPT/Ctrl classifier, 92.86% for SNPT/Ctrl classifier, 83.33% for SPPT/SNPT classifier; sensitivity: 80.00% for SPPT/Ctrl classifier, 85.71% for SNPT/Ctrl classifier, 80.00% for SPPT/SNPT classifier; specificity: 85.71% for SPPT/Ctrl classifier, 100% for SNPT/Ctrl classifier, 85.71% for SPPT/SNPT classifier; PPV: 80.00% for SPPT/Ctrl classifier, 100% for SNPT/Ctrl classifier, 80.00% for SPPT/SNPT classifier; NPV: 85.71% for SPPT/Ctrl classifier, 87.50% for SNPT/Ctrl classifier, 85.71% for SPPT/SNPT classifier; Table 3). In the SPPT/SNPT/Ctrl group, the optimized biomarker combination (F_4_: 9 features) could achieve higher three-class classification accuracy (89.47%), sensitivity (80%, 85.71% and 100% for SPPT, SNPT and control groups), specificity (100%, 91.67%, and 91.67% for SPPT, SNPT and control groups), PPV (100%, 85.71% and 87.50% for SPPT, SNPT and control groups) and NPV (93.33%, 91.67% and 100% for SPPT, SNPT and control groups) (Fig. 5). These results demonstrated good performance of the four feature sets (F_1_–F_4_) for precise identification of any one of the SPPT and SNPT patients and controls.

**Table 3:** Classification performance of binary classifications with selected feature combinations on test sets

	Accuracy	Sensitivity	Specificity	PPV	NPV
RF					
SPPT/Ctrl (F_1_)	83.33%	80.00%	85.71%	80.00%	85.71%
SNPT/Ctrl (F_2_)	92.86%	85.71%	100%	100%	87.50%
SPPT/SNPT (F_3_)	83.33%	80.00%	85.71%	80.00%	85.71%
SVM					
SPPT/Ctrl (F_1_)	91.67%	80.00%	100%	100%	87.50%
SNPT/Ctrl (F_2_)	92.86%	85.71%	100%	100%	87.50%
SPPT/SNPT (F_3_)	91.67%	80.00%	100%	100%	87.50%
MLP					
SPPT/Ctrl (F_1_)	83.33%	60.00%	100%	100%	77.78%
SNPT/Ctrl (F_2_)	92.86%	85.71%	100%	100%	87.50%
SPPT/SNPT (F_3_)	91.67%	80.00%	100%	100%	87.50%

F_1_ set: albumin and 9-OxoODE; F_2_ set: l-Pyroglutamic acid, Enterostatin human and 9-OxoODE; F_3_ set: Val-Ser, Methoxyacetic acid and Ethyl 3-hydroxybutyrate

The other two machine learning methods (SVM and MLP) were further adopted to verify the classification performance of the above-mentioned four biomarker combinations. As expected, the above four biomarker combinations showed high classification accuracy in SVM and MLP methods as that in RF method (Table 3, Fig. 5). Especially, compared with RF and SVM methods, MLP displayed the best classification performance (accuracy: 94.74%; sensitivity: 100%, 85.71% and 100% for SPPT, SNPT and control groups, specificity: 100%, 91.67%, and 100% for SPPT, SNPT and control groups, PPV: 100%, 100% and 87.50% for SPPT, SNPT and control groups and NPV: 100%, 92.31% and 100% for SPPT, SNPT and control groups) for simultaneously discriminating the SPPT and SNPT patients and controls (Fig. 5), indicating the potential in disease classification/diagnosis for MLP.

## Discussion

Our study revealed significantly enrichment of lipid/lipid-like molecules and organic acids/derivatives in the SPPT and SNPT patients, indicating the dysfunctional fatty acid and amino acid metabolisms, which is in agreement with previous reports [32–35]. Here, the SPPT samples showed a more serious dysfunction in fatty acid and amino acid metabolisms. Further, four promising diagnostic marker combinations (including nine lipid/lipid-like and organic acids/derivatives molecules and one clinical indicator) were screened out for precise classification of SPPT patients, SNPT patients and controls with high accuracy (83.33–92.86%): a lipid-like molecule combined with a clinical indicator (albumin and 9-OxoODE) could precisely differentiate SPPT patients from controls (accuracy: 83.33%); two lipid/lipid-like and one organic acid molecules (PGA, Enterostatin human and 9-OxoODE) could precisely distinguish SNPT patients from controls (accuracy: 92.86%); three organic acid molecules (Val-Ser, MAA and Ethyl 3-hydroxybutyrate) could precisely classify SPPT and SNPT patients (accuracy: 83.33%); two lipid/lipid-like and seven organic acid molecules (9-OxoODE, PGA, Val-Ser, Ethyl 3-hydroxybutyrate, MAA, Enterostatin human, DL-Norvaline, His-Pro and EPA) could simultaneously precise identify SPPT patients, SNPT patients and controls (accuracy: 89.47%).

As we know, lipids/lipid-like molecules are a type of important structural material of *Mtb*, especially in the bacterial cell wall [38], which possesses a rich repository of lipid remodeling enzymes to utilize host fatty acids for their survival in the harsh hypoxic microenvironment [39], further, resulting in serious dysfunctional lipid metabolism in TB patients [40]. For amino acid metabolism, since TB is a chronic consumptive disease, various types of amino acids and proteins are essential for *Mtb* to survive in the human body, thus leading to the dysfunctional amino acid metabolism for TB patients [32]. As expected, our study identified some significantly differential (up-/down-regulated) lipid and amino acid metabolites to precisely discriminate SPPT patients, SNPT patients and controls through machine learning methods. Certainly, these markers and panels warrant further confirmation and optimization with larger sample size studies.

The nine lipid/lipid-like and organic acids/derivatives molecules from four potential diagnostic biomarker combinations include two lipid/lipid-like molecules (9-OxoODE and EPA), and seven organic acids/derivatives (PGA, DL-Norvaline, MAA, His-Pro, Val-Ser, Ethyl 3-hydroxybutyrate and Enterostatin human) (Additional files 3, 4, 5).

First, the two lipid/lipid-like molecules show significant downregulation/inhibition in the SPPT and SNPT patients (Additional file 1: Fig. S5). Here, 9-OxoODE ranks the first, the first and the third in the classification biomarkers for SPPT/Ctrl, SPPT/SNPT/Ctrl and SNPT/Ctrl groups, respectively (Fig. 4). A previous study has shown that the significantly inhibited 9-OxoODE also reflects a negative regulation for lipolysis induced inflammatory response in SPPT and SNPT patients, since 9-OxoODE (metabolite of linoleic acid) can activate the lipogenic machinery as a ligand nuclear receptor in PPAR-α and PPAR-γ [41–44]. Another lipid/lipid-like molecule, EPA ranks seventh in the classification biomarkers for SPPT/SNPT/Ctrl (Fig. 4). Previous studies have reported that significantly downregulated EPA can result in dysfunctional inflammatory responses in TB patients by downregulating the pro-inflammatory cytokines and upregulating lipid synthesis of immune cells [45].

For the abovementioned seven organic acids/derivatives as potential classification biomarkers, compared with controls, three ones (PGA, MAA and DL-Norvaline) show significant downregulation and His-Pro shows significant upregulation in both SPPT and SNPT patients (Additional file 1: Fig. S5). Here, PGA ranks the first and fourth for the classification biomarkers for SNPT/Ctrl and SPPT/SNPT/Ctrl groups, respectively (Fig. 4). Significantly downregulated PGA has been reported to improve the *Mtb* growth by inhibiting the biosynthesis of glutathione in human bodies [46–49]. MAA ranks third and fifth among the classification biomarkers for SPPT/SNPT and SPPT/SNPT/Ctrl groups, respectively (Fig. 4). Significantly downregulated MAA could result in a poor inhibition of mPTPB essential for the survival of *Mtb*, since it has been shown to catalyze the formation of an inhibitor of a *Mycobacterium* protein (tyrosine phosphatase B: mPTPB) [50]. In addition, DL-Norvaline and His-Pro rank the eighth and ninth among the classification biomarkers for SPPT/SNPT/Ctrl group (Fig. 4), both of which showed similar expressed trends, suggesting the dysfunction in both SPPT and SNPT patients.

The remaining three organic acid biomarker molecules (Val-Ser, Ethyl 3-hydroxybutyrate and Enterostatin human) show differential enrichment between the SPPT and SNPT patients. Here Val-Ser and Ethyl 3-hydroxybutyrate show specifically downregulated and upregulated in SPPT patients, respectively (Additional file 1: Fig. S5). They rank the first and second among the features for the differentiation of SPPT/SNPT group, and rank the third and second among the features for the differentiation of SPPT/SNPT/Ctrl group, respectively (Fig. 4). “Enterostatin human” was specifically upregulated in SNPT patients, and ranks the second and sixth among the selected features for the differentiation of SNPT/Ctrl and SPPT/SNPT/Ctrl groups, respectively (Fig. 4). The three organic acids/derivatives with specific changes in only one group display unique feature for the classification of various types of TB patients.

In addition, a clinical indicator of albumin ranks second in the feature set for the differentiation of SPPT/Ctrl group, indicating the better precision diagnosis of SPPT patients through combining metabolome and clinical indicators (Fig. 4). Previous reports have indicated a prognostic marker of TB patients for albumin, which is a critical nutrient and inflammation related protein marker [51].

Our finding further shows the potential of machine learning in the precise diagnosis of SPPT and SNPT patients. Machine learning is becoming ubiquitous for analyzing multi-dimensional big data, and has been widely applied to many biological/medical fields, including diagnostic biomarker identification [52], therapeutic targets detection [53], disease progression prediction [54], and causal relationship between phenotype and genotype [55]. In our study, three machine learning methods are used to screen out potential biomarkers for precise classification of various types of TB from multidimensional data. RF was first adopted to screen out precise classification biomarkers, since it has been widely applied to classification and feature selection for big data; we then obtained some important classification features according to the ranks of variables and their predictive importance. Previous studies have also demonstrated the good performance of RF method for discriminating TB from Non-TB [56]. The other two machine learning methods (SVM and MLP) were further used to verify the classification accuracy of the biomarker combinations. SVM is an ensemble machine learning to improve classification performance compared with a single classifier, which has also been applied in the prediction of disease progression such as breast cancer [57]. MLP is very famous for its autonomic learning capacity without the requirement of previous knowledge, which has also been used in the diagnosis of TB [58] and assessment of prognostic risk for SNPT patients [59]. Our research indicated the best classification performance of MLP for simultaneously identifying the SPPT, SNPT, and controls, with the highest accuracy of 94.74%, suggesting the advantage of MLP over RF and SVM to some extent.

There are also some limitations in our study. Although we have included all the TB patients meeting the inclusion and exclusion criteria in the three hospitals during 2017–2018 (the Tuberculosis Prevention and Treatment Institute of Kashgar, the Second People’s Hospital of Aksu, and the Kuqa County Infectious Disease Hospital), this is indeed a limitation of our study for not calculating the needed sample size as epidemiological survey. The relatively small training and test sets may decrease the statistical power of the results, and this point warrants further confirmation and optimization with larger sample size studies in the future. In addition, we do not observe the impact of the demographic factors (age, occupation, BMI, etc.) on the metabolomic profiles (data not shown), but further confirmation with larger samples is also warranted. Certainly, to translate our classification model into clinical practice, many standardized works about data/workflow/sampling are still required. Overall, all binary and three-class classifiers obtained from our study showed good performance for precisely identifying SPPT, SNPT and Ctrl groups in spite of some limitations, and some classification biomarkers have also been reported to be closely associated with TB [45, 49, 50].

## Conclusions

Our current study not only screens out four biomarker combinations for precise detection of SPPT and SNPT patients through combining plasma metabolites with clinical indicators, but also shows promising application of machine learning on the identification of diagnostic biomarkers from multi-dimensional big data.

Over recent decades, despite the rapid advancement of various diagnostic technologies, diagnostic errors (missed, delayed, or wrong diagnoses) are still the most common problems for many important diseases, such as lung cancer [52]. Multi-omics and machine learning provide powerful tools for solving these problems, and researchers can achieve precise classifications/diagnoses for the misdiagnosed diseases through integrating multi-omics data with machine learning [15, 18, 52]. Our research presents a successful attempt to precisely detect various types of TBs by integrating multi-omics data with machine learning, and further provides a good example and workflow for future studies on the precision diagnosis of various misdiagnosed diseases.

## Supplementary Information

**Additional file 1.** Supplementary data.

**Additional file 2.** Correlation matrix of 120 features.

**Additional file 3.** Detailed information of the 70 DAMs between the SPPT patients and controls.

**Additional file 4.** Detailed information of the 79 DAMs between the SNPT patients and controls.

**Additional file 5.** Detailed information of the 33 DAMs between the SPPT and SNPT patients.

## Data Availability

All data generated or analyzed during this study are included in this published article and its supplementary information files, further inquiries can be directed to the corresponding authors. Metabolomics data have been deposited to the EMBL-EBI MetaboLights database with the identifier MTBLS3787 [60]. The data and code used for the analysis in this study are available on GitHub (https://github.com/ChenF-Lab/SPPT.git).

## Abbreviations

Ctrl: Control people
CV: Cross validation
DAMs: Differentially abundant metabolites
EPA: Eicosapentaenoic acid
ESR: Erythrocyte sedimentation rate
IQR: Interquartile range
KNN: K-nearest neighbor
MAA: Methoxyacetic acid
MDG: Mean decrease in Gini coefficient
MLP: Multilayer perceptron neural network
*Mtb*: *Mycobacterium tuberculosis*
OPLS-DA: Orthogonal partial least squares-discriminant analysis
PCA: Principal component analysis
PGA: L-pyroglutamic acid
RF: Random forest
SNPT: Smear-negative pulmonary tuberculosis
ROC: Receiver operating characteristic
SPPT: Smear-positive pulmonary tuberculosis
SVM: Support vector machines
TB: Tuberculosis
VIP: Variable importance in projection
