# Supplementary material for: Combining metabolome and clinical indicators with machine learning provides some promising diagnostic markers to precisely detect smear-positive/negative pulmonary tuberculosis
Source: BMC Infect Dis. 2022 Aug 25;22:707. doi: 10.1186/s12879-022-07694-8 (PMC9403968; doi:10.1186/s12879-022-07694-8)
Supplement: Supplementary file 1 — Additional file 1. Supplementary data. [file 12879_2022_7694_MOESM1_ESM.docx]

**Additional file 1. Supplementary data**

**Combining metabolome and clinical indicators with machine learning provides some promising diagnostic markers to precisely detect smear-positive/negative pulmonary tuberculosis**

Xin Hu^1,2†^, Jie Wang^3,4†^, Yingjiao Ju^3,4†^, Xiuli Zhang^3†^, Wushou’er Qimanguli^5^, Cuidan Li^3^, Liya Yue^3^, Bahetibieke Tuohetaerbaike^6^, Ying Li^6^, Hao Wen^6^, Wenbao Zhang^6^, Changbin Chen^7^, Yefeng Yang^8^, Jing Wang^8,1*^, Fei Chen^3,4,6*^

Table S1. Percentage of missing value in variables.

Table S2. The enriched metabolic pathways of the SPPT/Ctrl group.

Table S3. The enriched metabolic pathways of the SNPT/Ctrl group.

Table S4. The enriched metabolic pathways of the SPPT/SNPT group.

Table S5. Accuracy of 10-fold cross-validation for the four classifiers (78 features) on validation sets.

Table S6. Accuracy of 10-fold cross-validation for the 12 classifiers (10 features) on validation sets after feature selection.

Figure S1. The workflow of this study.

Figure S2. The top-10 contributed variables for PC1 and PC2 in the PCA of the combination of clinical indicators and DAMs (120 features).

Figure S3. Confusion matrixes and ROC curves for classifying SPPT, SNPT and controls through RF using 78 features.

Figure S4. Screening workflow for optimized biomarkers to precisely distinguish SPPT, SNPT patients and controls.

Figure S5. Box plots showing the abundances of 10 optimized clinical/metabolic features for SPPT, SNPT patients and controls.

**Table S1. Percentage of missing value in variables**

| **Variables** | **Missing (N)** | **Missing (%)** |
| --- | --- | --- |
| Procalcitonin | 51 | 51 |
| Treatment | 42 | 42 |
| Indirect bilirubin | 42 | 42 |
| Contact | 40 | 40 |
| C-reaction protein | 40 | 40 |
| ESR | 39 | 39 |
| Cavitary pulmonary TB | 36 | 36 |
| Symptoms | 36 | 36 |
| Direct bilirubin | 30 | 30 |
| Occupation | 25 | 25 |
| Neutrophils | 17 | 17 |
| BMI | 16 | 16 |
| Globulin | 13 | 13 |
| Smoking status | 9 | 9 |
| Drinking status | 8 | 8 |
| Triglyceride | 5 | 5 |
| TC | 5 | 5 |
| AKP | 4 | 4 |
| γ-GT | 4 | 4 |
| Total protein | 2 | 2 |
| Albumin | 2 | 2 |
| Total bilirubin | 2 | 2 |
| Marital status | 1 | 1 |
| Platelets | 1 | 1 |
| ASP | 1 | 1 |
| ALT | 1 | 1 |
| Gender | 0 | 0 |
| Age | 0 | 0 |
| Leucocytes | 0 | 0 |
| Erythrocytes | 0 | 0 |
| Hemoglobin | 0 | 0 |
| Eosinophils | 0 | 0 |
| Basophils | 0 | 0 |
| Creatinine | 0 | 0 |
| (4Z,7Z,10Z,13Z,16Z,19Z)-4,7,10,13,1 6,19-Docosahexaenoic acid | 0 | 0 |
| 1-Oleoyl-sn-glycero-3-phosphocholine | 0 | 0 |
| 1-Palmitoyl-sn-glycero-3-phosphocholine | 0 | 0 |
| 1-Stearoyl-2-hydroxy-sn-glycero-3-phosphocholine | 0 | 0 |
| 1,2-dioleoyl-sn-glycero-3-phosphatidylcholine | 0 | 0 |
| 11(Z),14(Z)-Eicosadienoic Acid | 0 | 0 |
| 16-Hydroxypalmitic acid | 0 | 0 |
| 2-Ethoxyethanol | 0 | 0 |
| 2-Ethyl-2-Hydroxybutyric acid | 0 | 0 |
| 2-Hydroxy-3-methylbutyric acid | 0 | 0 |
| 2-Methylglutaric acid | 0 | 0 |
| 3-Hydroxypropionic acid (beta-lactic acid) | 0 | 0 |
| 4-Pyridinecarboxylic acid | 0 | 0 |
| 9-OxoODE | 0 | 0 |
| 9R,10S-EpOME | 0 | 0 |
| Acetylcarnitine | 0 | 0 |
| Acetylglycine | 0 | 0 |
| all cis-(6,9,12)-Linolenic acid | 0 | 0 |
| Anthranilic acid (Vitamin L1) | 0 | 0 |
| Arachidic acid | 0 | 0 |
| Benzoic acid | 0 | 0 |
| Betaine | 0 | 0 |
| Caffeine | 0 | 0 |
| Capric acid | 0 | 0 |
| Chenodeoxycholate | 0 | 0 |
| Choline | 0 | 0 |
| cis-9-Palmitoleic acid | 0 | 0 |
| Citrate | 0 | 0 |
| Creatinine | 0 | 0 |
| D-Allose | 0 | 0 |
| D-erythro-Sphingosine-1-phosphate | 0 | 0 |
| D-Mannose | 0 | 0 |
| D-Pipecolinic acid | 0 | 0 |
| D-Proline | 0 | 0 |
| D-Quinovose | 0 | 0 |
| D-Tagatose | 0 | 0 |
| D-Threitol | 0 | 0 |
| Decanoyl-L-carnitine | 0 | 0 |
| Dihydroxyacetone | 0 | 0 |
| Dimethylglycine | 0 | 0 |
| DL-3-Phenyllactic acid | 0 | 0 |
| DL-Indole-3-lactic acid | 0 | 0 |
| DL-lactate | 0 | 0 |
| DL-Norvaline | 0 | 0 |
| Docosatrienoic Acid | 0 | 0 |
| Dodecanoic acid | 0 | 0 |
| Eicosapentaenoic acid | 0 | 0 |
| Enterostatin human | 0 | 0 |
| Ergothioneine | 0 | 0 |
| Ethyl 3-hydroxybutyrate | 0 | 0 |
| Glycerophosphocholine | 0 | 0 |
| Glycochenodeoxycholate | 0 | 0 |
| Glycocholic acid | 0 | 0 |
| His-Pro | 0 | 0 |
| Hydroxyisocaproic acid | 0 | 0 |
| Hypoxanthine | 0 | 0 |
| ketoisocaproic acid | 0 | 0 |
| L-(-)Sorbose | 0 | 0 |
| L-Arginine | 0 | 0 |
| L-Fucose | 0 | 0 |
| L-Glutamine | 0 | 0 |
| L-Gulonic gamma-lactone | 0 | 0 |
| L-Histidine | 0 | 0 |
| L-Leucine | 0 | 0 |
| L-Palmitoylcarnitine | 0 | 0 |
| L-Pyroglutamic acid | 0 | 0 |
| L-Rhamnose | 0 | 0 |
| L-Tryptophan | 0 | 0 |
| Linoleic acid | 0 | 0 |
| Methoxyacetic acid | 0 | 0 |
| Myristic acid | 0 | 0 |
| N-Oleoylethanolamine | 0 | 0 |
| N1-Methyl-2-pyridone-5-carboxamide | 0 | 0 |
| Nicotinate | 0 | 0 |
| Nname,cis-9,10-Epoxystearic acid | 0 | 0 |
| Palmitic acid | 0 | 0 |
| PC(16:0/16:0) | 0 | 0 |
| Pentadecanoic Acid | 0 | 0 |
| Pimelic acid | 0 | 0 |
| Pseudouridine | 0 | 0 |
| Stearic acid | 0 | 0 |
| Stearidonic Acid | 0 | 0 |
| Taurochenodeoxycholate | 0 | 0 |
| Tetrahydrocorticosterone | 0 | 0 |
| Theophylline | 0 | 0 |
| trans-Vaccenic acid | 0 | 0 |
| Trimethylamine N-oxide | 0 | 0 |
| Urea | 0 | 0 |
| Uric acid | 0 | 0 |
| Val-Ser | 0 | 0 |
| Val-Tyr | 0 | 0 |
| L-Pipecolic acid | 0 | 0 |
| Maslinic Acid | 0 | 0 |
| Hydrocortisone (Cortisol) | 0 | 0 |
| L-Carnitine | 0 | 0 |
| Undecanoic Acid | 0 | 0 |

**Table S2. The enriched metabolic pathways of the SPPT/Ctrl group**

| **Pathways** | **Total** | **Expected** | **Hits** | **Raw p** | **-log10(p)** | **Holm adjust** | **FDR** | **Impact** |
| --- | --- | --- | --- | --- | --- | --- | --- | --- |
| Biosynthesis of unsaturated fatty acids | 36.00 | 1.35 | 6.00 | 0.00 | 2.76 | 0.14 | 0.14 | 0.00 |
| Linoleic acid metabolism | 5.00 | 0.19 | 2.00 | 0.01 | 1.89 | 1.00 | 0.54 | 1.00 |
| Valine, leucine and isoleucine biosynthesis | 8.00 | 0.30 | 2.00 | 0.03 | 1.48 | 1.00 | 0.93 | 0.00 |
| Primary bile acid biosynthesis | 46.00 | 1.72 | 4.00 | 0.09 | 1.05 | 1.00 | 1.00 | 0.03 |
| Arginine biosynthesis | 14.00 | 0.52 | 2.00 | 0.09 | 1.03 | 1.00 | 1.00 | 0.00 |
| Fatty acid biosynthesis | 47.00 | 1.76 | 4.00 | 0.10 | 1.02 | 1.00 | 1.00 | 0.01 |
| Aminoacyl-tRNA biosynthesis | 48.00 | 1.80 | 4.00 | 0.10 | 0.99 | 1.00 | 1.00 | 0.00 |
| Nicotinate and nicotinamide metabolism | 15.00 | 0.56 | 2.00 | 0.11 | 0.98 | 1.00 | 1.00 | 0.00 |
| beta-Alanine metabolism | 21.00 | 0.79 | 2.00 | 0.18 | 0.74 | 1.00 | 1.00 | 0.00 |
| D-Glutamine and D-glutamate metabolism | 6.00 | 0.22 | 1.00 | 0.20 | 0.69 | 1.00 | 1.00 | 0.00 |
| Nitrogen metabolism | 6.00 | 0.22 | 1.00 | 0.20 | 0.69 | 1.00 | 1.00 | 0.00 |
| Purine metabolism | 65.00 | 2.43 | 4.00 | 0.22 | 0.65 | 1.00 | 1.00 | 0.02 |
| Ascorbate and aldarate metabolism | 8.00 | 0.30 | 1.00 | 0.26 | 0.58 | 1.00 | 1.00 | 0.00 |
| Alanine, aspartate and glutamate metabolism | 28.00 | 1.05 | 2.00 | 0.28 | 0.55 | 1.00 | 1.00 | 0.11 |
| Glyoxylate and dicarboxylate metabolism | 32.00 | 1.20 | 2.00 | 0.34 | 0.47 | 1.00 | 1.00 | 0.03 |
| Glycerophospholipid metabolism | 36.00 | 1.35 | 2.00 | 0.39 | 0.41 | 1.00 | 1.00 | 0.07 |
| Fatty acid degradation | 39.00 | 1.46 | 2.00 | 0.43 | 0.36 | 1.00 | 1.00 | 0.00 |
| Valine, leucine and isoleucine degradation | 40.00 | 1.50 | 2.00 | 0.45 | 0.35 | 1.00 | 1.00 | 0.01 |
| Glycerolipid metabolism | 16.00 | 0.60 | 1.00 | 0.46 | 0.34 | 1.00 | 1.00 | 0.00 |
| Histidine metabolism | 16.00 | 0.60 | 1.00 | 0.46 | 0.34 | 1.00 | 1.00 | 0.22 |
| Fructose and mannose metabolism | 20.00 | 0.75 | 1.00 | 0.54 | 0.27 | 1.00 | 1.00 | 0.00 |
| Ether lipid metabolism | 20.00 | 0.75 | 1.00 | 0.54 | 0.27 | 1.00 | 1.00 | 0.00 |
| Citrate cycle (TCA cycle) | 20.00 | 0.75 | 1.00 | 0.54 | 0.27 | 1.00 | 1.00 | 0.09 |
| Pyruvate metabolism | 22.00 | 0.82 | 1.00 | 0.57 | 0.24 | 1.00 | 1.00 | 0.00 |
| Propanoate metabolism | 23.00 | 0.86 | 1.00 | 0.59 | 0.23 | 1.00 | 1.00 | 0.00 |
| Glycolysis / Gluconeogenesis | 26.00 | 0.97 | 1.00 | 0.63 | 0.20 | 1.00 | 1.00 | 0.00 |
| Glycine, serine and threonine metabolism | 33.00 | 1.23 | 1.00 | 0.72 | 0.14 | 1.00 | 1.00 | 0.05 |
| Amino sugar and nucleotide sugar metabolism | 37.00 | 1.38 | 1.00 | 0.76 | 0.12 | 1.00 | 1.00 | 0.00 |
| Arginine and proline metabolism | 38.00 | 1.42 | 1.00 | 0.77 | 0.11 | 1.00 | 1.00 | 0.00 |
| Fatty acid elongation | 39.00 | 1.46 | 1.00 | 0.78 | 0.11 | 1.00 | 1.00 | 0.00 |
| Pyrimidine metabolism | 39.00 | 1.46 | 1.00 | 0.78 | 0.11 | 1.00 | 1.00 | 0.00 |
| Drug metabolism - other enzymes | 39.00 | 1.46 | 1.00 | 0.78 | 0.11 | 1.00 | 1.00 | 0.00 |
| Tryptophan metabolism | 41.00 | 1.53 | 1.00 | 0.79 | 0.10 | 1.00 | 1.00 | 0.14 |

**Table S3. The enriched metabolic pathways of the SNPT/Ctrl group**

| **Pathways** | **Total** | **Expected** | **Hits** | **Raw p** | **-log10(p)** | **Holm adjust** | **FDR** | **Impact** |
| --- | --- | --- | --- | --- | --- | --- | --- | --- |
| Biosynthesis of unsaturated fatty acids | 36.00 | 1.49 | 7.00 | 0.00 | 3.32 | 0.04 | 0.04 | 0.00 |
| Linoleic acid metabolism | 5.00 | 0.21 | 2.00 | 0.02 | 1.81 | 1.00 | 0.65 | 1.00 |
| Glycerophospholipid metabolism | 36.00 | 1.49 | 4.00 | 0.06 | 1.24 | 1.00 | 1.00 | 0.19 |
| alpha-Linolenic acid metabolism | 13.00 | 0.54 | 2.00 | 0.10 | 1.01 | 1.00 | 1.00 | 0.00 |
| Nicotinate and nicotinamide metabolism | 15.00 | 0.62 | 2.00 | 0.12 | 0.90 | 1.00 | 1.00 | 0.00 |
| Fatty acid biosynthesis | 47.00 | 1.94 | 4.00 | 0.13 | 0.90 | 1.00 | 1.00 | 0.01 |
| Glycine, serine and threonine metabolism | 33.00 | 1.36 | 3.00 | 0.15 | 0.82 | 1.00 | 1.00 | 0.12 |
| beta-Alanine metabolism | 21.00 | 0.87 | 2.00 | 0.21 | 0.67 | 1.00 | 1.00 | 0.00 |
| Ascorbate and aldarate metabolism | 8.00 | 0.33 | 1.00 | 0.29 | 0.54 | 1.00 | 1.00 | 0.00 |
| Valine, leucine and isoleucine biosynthesis | 8.00 | 0.33 | 1.00 | 0.29 | 0.54 | 1.00 | 1.00 | 0.00 |
| Caffeine metabolism | 10.00 | 0.41 | 1.00 | 0.34 | 0.46 | 1.00 | 1.00 | 0.00 |
| Arginine biosynthesis | 14.00 | 0.58 | 1.00 | 0.45 | 0.35 | 1.00 | 1.00 | 0.08 |
| Arginine and proline metabolism | 38.00 | 1.57 | 2.00 | 0.47 | 0.33 | 1.00 | 1.00 | 0.06 |
| Fatty acid degradation | 39.00 | 1.61 | 2.00 | 0.48 | 0.31 | 1.00 | 1.00 | 0.00 |
| Histidine metabolism | 16.00 | 0.66 | 1.00 | 0.49 | 0.31 | 1.00 | 1.00 | 0.22 |
| Fructose and mannose metabolism | 20.00 | 0.83 | 1.00 | 0.57 | 0.24 | 1.00 | 1.00 | 0.00 |
| Ether lipid metabolism | 20.00 | 0.83 | 1.00 | 0.57 | 0.24 | 1.00 | 1.00 | 0.00 |
| Sphingolipid metabolism | 21.00 | 0.87 | 1.00 | 0.59 | 0.23 | 1.00 | 1.00 | 0.02 |
| Aminoacyl-tRNA biosynthesis | 48.00 | 1.98 | 2.00 | 0.60 | 0.22 | 1.00 | 1.00 | 0.00 |
| Pyruvate metabolism | 22.00 | 0.91 | 1.00 | 0.61 | 0.22 | 1.00 | 1.00 | 0.00 |
| Propanoate metabolism | 23.00 | 0.95 | 1.00 | 0.62 | 0.21 | 1.00 | 1.00 | 0.00 |
| Glycolysis / Gluconeogenesis | 26.00 | 1.07 | 1.00 | 0.67 | 0.17 | 1.00 | 1.00 | 0.00 |
| Glutathione metabolism | 28.00 | 1.16 | 1.00 | 0.70 | 0.16 | 1.00 | 1.00 | 0.01 |
| Purine metabolism | 65.00 | 2.68 | 2.00 | 0.76 | 0.12 | 1.00 | 1.00 | 0.02 |
| Arachidonic acid metabolism | 36.00 | 1.49 | 1.00 | 0.78 | 0.11 | 1.00 | 1.00 | 0.00 |
| Amino sugar and nucleotide sugar metabolism | 37.00 | 1.53 | 1.00 | 0.79 | 0.10 | 1.00 | 1.00 | 0.00 |
| Fatty acid elongation | 39.00 | 1.61 | 1.00 | 0.81 | 0.09 | 1.00 | 1.00 | 0.00 |
| Drug metabolism - other enzymes | 39.00 | 1.61 | 1.00 | 0.81 | 0.09 | 1.00 | 1.00 | 0.00 |
| Valine, leucine and isoleucine degradation | 40.00 | 1.65 | 1.00 | 0.82 | 0.09 | 1.00 | 1.00 | 0.01 |
| Tryptophan metabolism | 41.00 | 1.69 | 1.00 | 0.83 | 0.08 | 1.00 | 1.00 | 0.00 |
| Primary bile acid biosynthesis | 46.00 | 1.90 | 1.00 | 0.86 | 0.07 | 1.00 | 1.00 | 0.00 |
| Steroid hormone biosynthesis | 85.00 | 3.51 | 1.00 | 0.97 | 0.01 | 1.00 | 1.00 | 0.00 |

**Table S4. The enriched metabolic pathways of the SPPT/SNPT group**

| **Pathways** | **Total** | **Expected** | **Hits** | **Raw p** | **-log10(p)** | **Holm adjust** | **FDR** | **Impact** |
| --- | --- | --- | --- | --- | --- | --- | --- | --- |
| Linoleic acid metabolism | 5.00 | 0.08 | 2.00 | 0.00 | 2.62 | 0.20 | 0.20 | 1.00 |
| alpha-Linolenic acid metabolism | 13.00 | 0.21 | 2.00 | 0.02 | 1.76 | 1.00 | 0.39 | 0.00 |
| Biosynthesis of unsaturated fatty acids | 36.00 | 0.58 | 3.00 | 0.02 | 1.73 | 1.00 | 0.39 | 0.00 |
| Glycerophospholipid metabolism | 36.00 | 0.58 | 3.00 | 0.02 | 1.73 | 1.00 | 0.39 | 0.17 |
| Primary bile acid biosynthesis | 46.00 | 0.74 | 2.00 | 0.17 | 0.77 | 1.00 | 1.00 | 0.01 |
| Arginine biosynthesis | 14.00 | 0.23 | 1.00 | 0.20 | 0.69 | 1.00 | 1.00 | 0.00 |
| Nicotinate and nicotinamide metabolism | 15.00 | 0.24 | 1.00 | 0.22 | 0.66 | 1.00 | 1.00 | 0.00 |
| Ether lipid metabolism | 20.00 | 0.32 | 1.00 | 0.28 | 0.55 | 1.00 | 1.00 | 0.00 |
| Citrate cycle (TCA cycle) | 20.00 | 0.32 | 1.00 | 0.28 | 0.55 | 1.00 | 1.00 | 0.09 |
| Lysine degradation | 25.00 | 0.40 | 1.00 | 0.34 | 0.47 | 1.00 | 1.00 | 0.00 |
| Alanine, aspartate and glutamate metabolism | 28.00 | 0.45 | 1.00 | 0.37 | 0.43 | 1.00 | 1.00 | 0.00 |
| Steroid hormone biosynthesis | 85.00 | 1.37 | 2.00 | 0.40 | 0.40 | 1.00 | 1.00 | 0.03 |
| Glyoxylate and dicarboxylate metabolism | 32.00 | 0.52 | 1.00 | 0.41 | 0.39 | 1.00 | 1.00 | 0.03 |
| Glycine, serine and threonine metabolism | 33.00 | 0.53 | 1.00 | 0.42 | 0.38 | 1.00 | 1.00 | 0.00 |
| Arachidonic acid metabolism | 36.00 | 0.58 | 1.00 | 0.45 | 0.35 | 1.00 | 1.00 | 0.00 |
| Drug metabolism - other enzymes | 39.00 | 0.63 | 1.00 | 0.47 | 0.32 | 1.00 | 1.00 | 0.00 |
| Fatty acid biosynthesis | 47.00 | 0.76 | 1.00 | 0.54 | 0.27 | 1.00 | 1.00 | 0.00 |
| Purine metabolism | 65.00 | 1.05 | 1.00 | 0.66 | 0.18 | 1.00 | 1.00 | 0.00 |

**Table S5. Accuracy of 10-fold cross-validation for the four classifiers (78 features) on validation sets**

| **Fold** | **Binary classification** | | |  | **Three-class classification** |
| --- | --- | --- | --- | --- | --- |
|  | **Accuracy (SPPT/Ctrl)** | **Accuracy (SNPT/Ctrl)** | **Accuracy (SPPT/SNPT)** |  | **Accuracy (SPPT/SNPT/Ctrl)** |
|  |  |  |  |  |  |
| 1 | 1.00 | 1.00 | 1.00 |  | 1.00 |
| 2 | 1.00 | 1.00 | 0.80 |  | 1.00 |
| 3 | 1.00 | 1.00 | 1.00 |  | 0.71 |
| 4 | 1.00 | 1.00 | 1.00 |  | 0.88 |
| 5 | 1.00 | 1.00 | 0.80 |  | 1.00 |
| 6 | 1.00 | 1.00 | 1.00 |  | 1.00 |
| 7 | 1.00 | 1.00 | 1.00 |  | 1.00 |
| 8 | 0.80 | 1.00 | 1.00 |  | 1.00 |
| 9 | 1.00 | 1.00 | 0.83 |  | 0.89 |
| 10 | 1.00 | 1.00 | 0.80 |  | 1.00 |
| **Mean** | 0.98 | 1.00 | 0.92 |  | 0.95 |
| **SD** | 0.06 | 0.00 | 0.09 |  | 0.09 |

**Table S6. Accuracy of 10-fold cross-validation for the 12 classifiers (10 features) on validation sets after feature selection**

| **Fold** | **RF** | | | |  | **SVM** | | | |  | **MLP** | | | |
| --- | --- | --- | --- | --- | --- | --- | --- | --- | --- | --- | --- | --- | --- | --- |
|  | **Accuracy (SPPT/**  **Ctrl)** | **Accuracy (SNPT/**  **Ctrl)** | **Accuracy (SPPT/**  **SNPT)** | **Accuracy (SPPT/SNPT/**  **Ctrl)** |  | **Accuracy (SPPT/**  **Ctrl)** | **Accuracy (SNPT/**  **Ctrl)** | **Accuracy (SPPT/**  **SNPT)** | **Accuracy (SPPT/SNPT/**  **Ctrl)** |  | **Accuracy (SPPT/**  **Ctrl)** | **Accuracy (SNPT/**  **Ctrl)** | **Accuracy (SPPT/**  **SNPT)** | **Accuracy (SPPT/SNPT**  **Ctrl)** |
|  |  |  |  |  |  |  |  |  |  |  |  |  |  |  |
| 1 | 1.00 | 1.00 | 1.00 | 1.00 |  | 1.00 | 1.00 | 0.80 | 1.00 |  | 1.00 | 1.00 | 1.00 | 0.89 |
| 2 | 1.00 | 1.00 | 1.00 | 1.00 |  | 1.00 | 1.00 | 1.00 | 1.00 |  | 1.00 | 1.00 | 0.83 | 0.75 |
| 3 | 0.80 | 1.00 | 0.80 | 0.71 |  | 1.00 | 1.00 | 0.60 | 0.88 |  | 0.80 | 1.00 | 1.00 | 1.00 |
| 4 | 1.00 | 1.00 | 1.00 | 0.88 |  | 1.00 | 1.00 | 1.00 | 1.00 |  | 1.00 | 1.00 | 1.00 | 1.00 |
| 5 | 1.00 | 0.83 | 0.80 | 0.88 |  | 1.00 | 1.00 | 0.83 | 1.00 |  | 0.80 | 1.00 | 0.80 | 0.88 |
| 6 | 1.00 | 1.00 | 1.00 | 1.00 |  | 1.00 | 1.00 | 1.00 | 0.88 |  | 1.00 | 1.00 | 1.00 | 1.00 |
| 7 | 1.00 | 0.83 | 1.00 | 1.00 |  | 0.80 | 1.00 | 0.80 | 1.00 |  | 1.00 | 1.00 | 0.80 | 0.88 |
| 8 | 1.00 | 1.00 | 1.00 | 0.88 |  | 1.00 | 1.00 | 0.80 | 1.00 |  | 1.00 | 1.00 | 0.80 | 0.75 |
| 9 | 0.80 | 1.00 | 0.67 | 0.89 |  | 1.00 | 1.00 | 1.00 | 0.75 |  | 0.80 | 1.00 | 1.00 | 1.00 |
| 10 | 1.00 | 1.00 | 1.00 | 1.00 |  | 1.00 | 1.00 | 0.83 | 0.89 |  | 1.00 | 1.00 | 1.00 | 0.88 |
| **Mean** | 0.96 | 0.97 | 0.93 | 0.92 |  | 0.98 | 1.00 | 0.87 | 0.94 |  | 0.94 | 1.00 | 0.92 | 0.90 |
| **SD** | 0.08 | 0.07 | 0.12 | 0.09 |  | 0.06 | 0.00 | 0.13 | 0.08 |  | 0.09 | 0.00 | 0.09 | 0.09 |


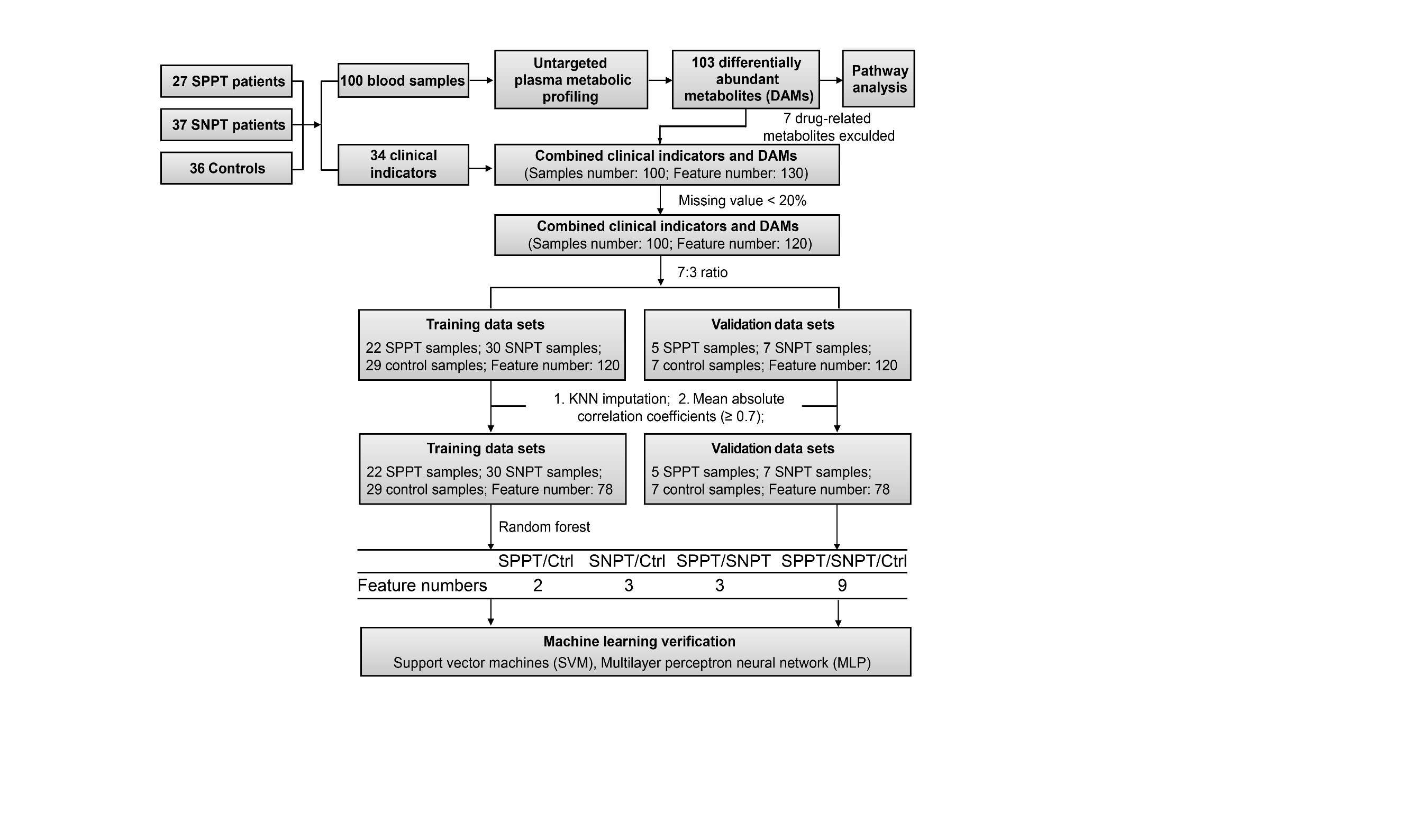


**Figure S1. The workflow of this study.**


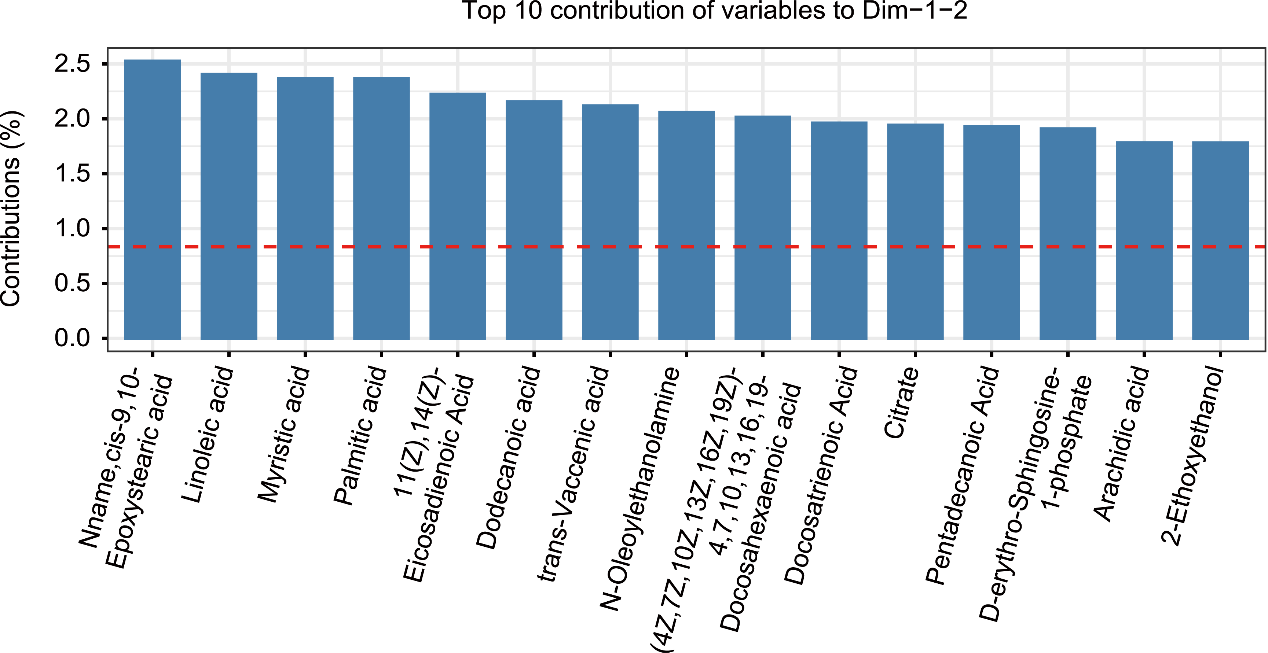


**Figure S2. The top-10 contributed variables for PC1 and PC2 in the PCA of the combination of clinical indicators and DAMs (120 features).**


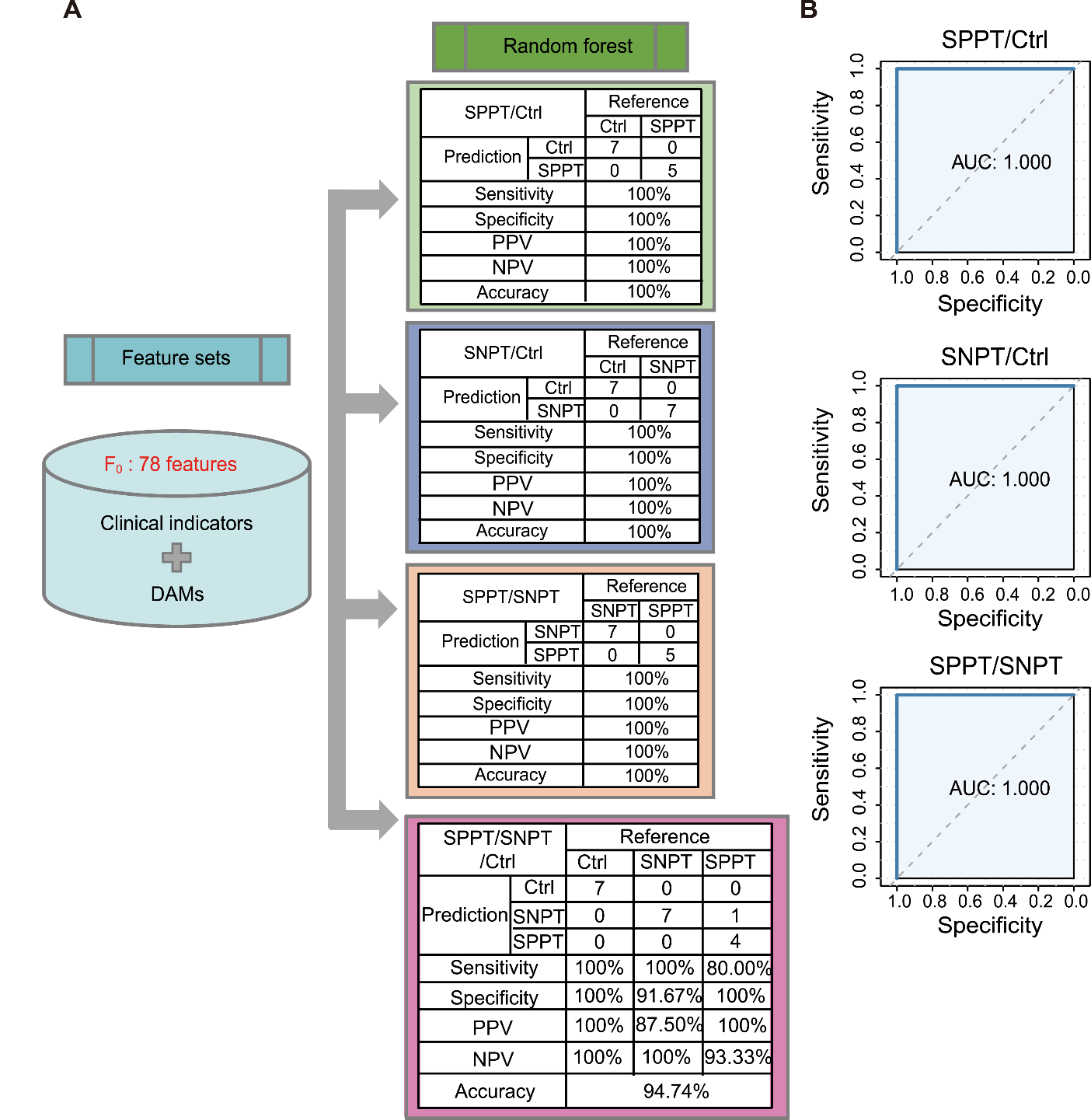


**Figure S3. Confusion matrixes and ROC curves for classifying SPPT, SNPT and controls through RF using 78 features.** (A) Confusion matrixes showing the SPPT/Ctrl, SNPT/Ctrl, SPPT/SNPT and SPPT/SNPT/Ctrl classifications in the test sets. (B) ROC curves showing the SPPT/Ctrl, SNPT/Ctrl and SPPT/SNPT classification performances in the test sets.


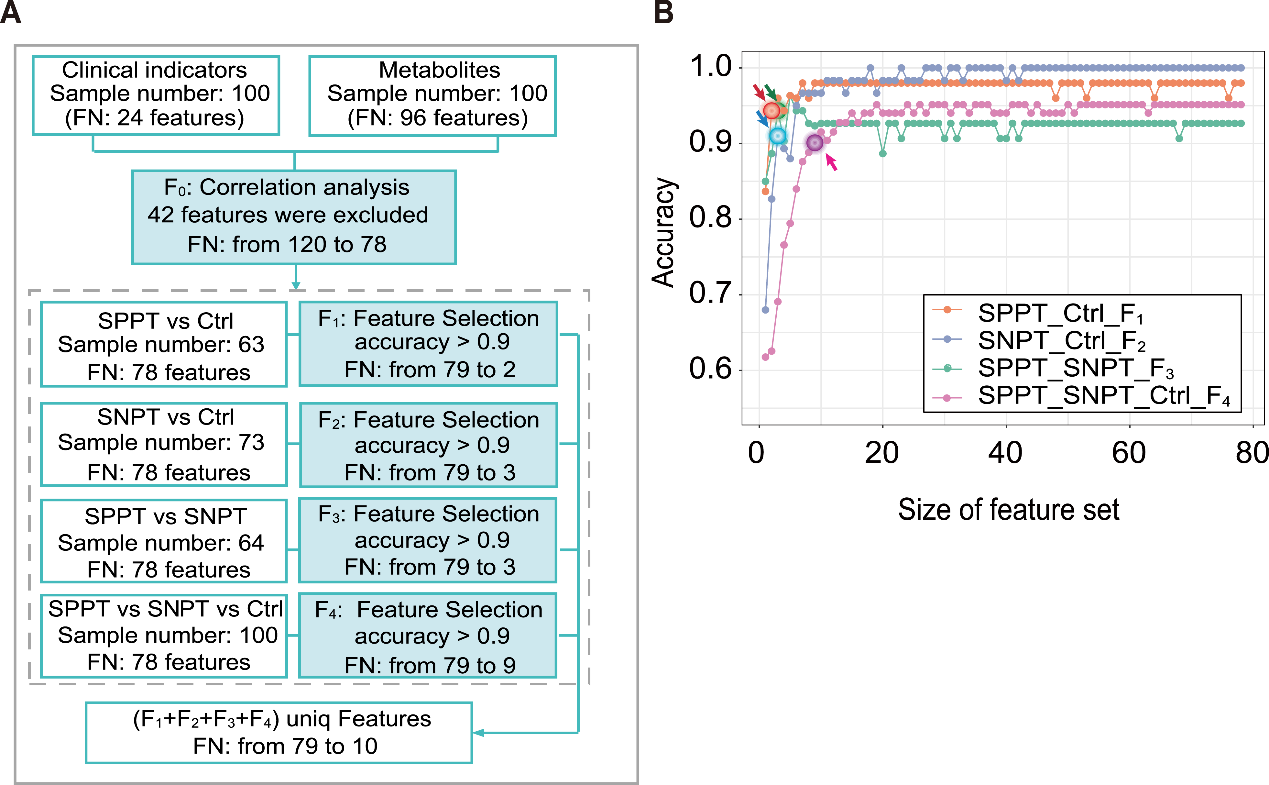


**Figure S4. Screening workflow for optimized biomarkers to precisely distinguish SPPT, SNPT patients and controls.** (A) Schematic showing the optimized feature selection process for binary and three-class classifications among the three groups. The selected optimized features were determined by higher accuracy (> 0.9) in random forest model. FN: the number of features. (B) The point plot displaying the increased accuracy with increase in feature number. The x-axis represents the number of features; the y-axis represents the accuracy of the RF model. Colored arrows indicate the selected optimized feature number.


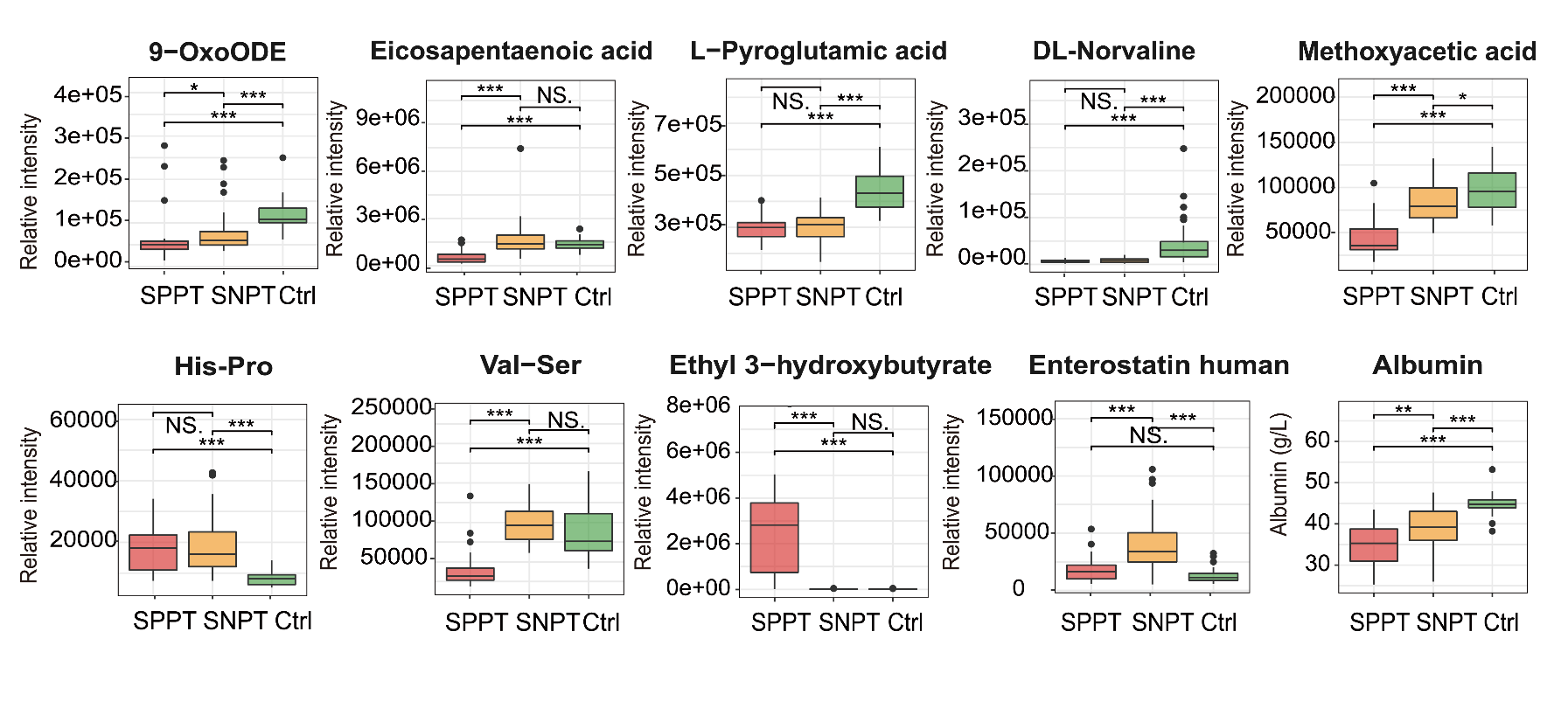


**Figure S5. Box plots showing the abundances of 10 optimized clinical/metabolic features for SPPT, SNPT patients and controls.** Differences between groups are estimated using Wilcoxon-test. *P < 0.05, **P < 0.01, ***P < 0.001. The y-axis represents the abundances of these selected features.
